# Pharmacology and Molecular Mechanisms of Clinically Relevant Estrogen Estetrol and Estrogen Mimic BMI-135 for the Treatment of Endocrine-Resistant Breast Cancer[Fn FN4]

**DOI:** 10.1124/molpharm.120.000054

**Published:** 2020-10

**Authors:** Balkees Abderrahman, Philipp Y. Maximov, Ramona F. Curpan, Jay S. Hanspal, Ping Fan, Rui Xiong, Debra A. Tonetti, Gregory R.J. Thatcher, V. Craig Jordan

**Affiliations:** Department of Breast Medical Oncology, University of Texas MD Anderson Cancer Center, Houston, Texas (B.A., P.Y.M., J.S.H., P.F., V.C.J.); Coriolan Dragulescu Institute of Chemistry, Romanian Academy, Timisoara, Romania (R.F.C.); and Department of Pharmaceutical Sciences, University of Illinois at Chicago, Chicago, Illinois (R.X., D.A.T., G.R.J.T.)

## Abstract

Long-term estrogen deprivation (LTED) with tamoxifen (TAM) or aromatase inhibitors leads to endocrine-resistance, whereby physiologic levels of estrogen kill breast cancer (BC). Estrogen therapy is effective in treating patients with advanced BC after resistance to TAM and aromatase inhibitors develops. This therapeutic effect is attributed to estrogen-induced apoptosis via the estrogen receptor (ER). Estrogen therapy can have unpleasant gynecologic and nongynecologic adverse events. Here, we study estetrol (E_4_) and a model Selective Human ER Partial Agonist (ShERPA) BMI-135. Estetrol and ShERPA TTC-352 are being evaluated in clinical trials. These agents are proposed as safer estrogenic candidates compared with 17*β*-estradiol (E_2_) for the treatment of endocrine-resistant BC. Cell viability assays, real-time polymerase chain reaction, luciferase reporter assays, chromatin immunoprecipitation, docking and molecular dynamics simulations, human unfolded protein response (UPR) RT^2^ PCR profiler arrays, live cell microscopic imaging and analysis, and annexin V staining assays were conducted. Our work was done in eight biologically different human BC cell lines and one human endometrial cancer cell line, and results were compared with full agonists estrone, E_2_, and estriol, a benchmark partial agonist triphenylethylene bisphenol (BPTPE), and antagonists 4-hydroxytamoxifen and endoxifen. Our study shows the pharmacology of E_4_ and BMI-135 as less-potent full-estrogen agonists as well as their molecular mechanisms of tumor regression in LTED BC through triggering a rapid UPR and apoptosis. Our work concludes that the use of a full agonist to treat BC is potentially superior to a partial agonist given BPTPE’s delayed induction of UPR and apoptosis, with a higher probability of tumor clonal evolution and resistance.

**SIGNIFICANCE STATEMENT:**

Given the unpleasant gynecologic and nongynecologic adverse effects of estrogen treatment, the development of safer estrogens for endocrine-resistant breast cancer (BC) treatment and hormone replacement therapy remains a priority. The naturally occurring estrogen estetrol and Selective Human Estrogen-Receptor Partial Agonists are being evaluated in endocrine-resistant BC clinical trials. This work provides a comprehensive evaluation of their pharmacology in numerous endocrine-resistant BC models and an endometrial cancer model and their molecular mechanisms of tumor regression through the unfolded protein response and apoptosis.

## Introduction

In 1944, Sir Alexander Haddow used high-dose synthetic estrogen therapy to treat metastatic breast cancer (MBC) (Haddow et al., 1944) in patients who were long-term (≥5 years past menopause) estrogen-deprived (LTED) (Haddow, 1970). A 30% response rate was reported. High-dose estrogen therapy was used for 30 years prior to the introduction of tamoxifen (TAM) (Jordan, 2003). Tamoxifen was preferred because of the lower incidence of adverse events (AEs) (Cole et al., 1971; Ingle et al., 1981). In the 1970s, the translational research proposal of long-term adjuvant antihormone TAM therapy was successfully advanced (Jordan et al., 1979; Jordan and Allen, 1980). This strategy established TAM as the agent of choice for adjuvant therapy (Early Breast Cancer Trialists’ Collaborative Group, 1998).

Acquired resistance to TAM therapy in vivo initially involves the growth of breast cancer (BC) populations within 1 to 2 years that are TAM- and estrogen-dependent (Gottardis and Jordan, 1988; Gottardis et al., 1989b). Subsequent studies in vivo demonstrated that 5 years of TAM treatment (mimicking the standard of care period at the time) leads to new BC populations that grow with TAM but die with physiologic levels of estrogen (Wolf and Jordan, 1993; Yao et al., 2000). This discovery explained (Jordan, 2008) why high-dose estrogen therapy was only effective ≥5 years past menopause in Haddow’s original clinical studies (Haddow, 1970).

Physiologic estrogen in LTED BC cells triggers a cellular stress response named the unfolded protein response (UPR) and induces apoptosis (Song et al., 2001; Lewis et al., 2005a; Ariazi et al., 2011). Hosford et al. (2019) confirmed the involvement of the UPR and apoptosis in patient-derived estrogen-deprived estrogen receptor (ER)-positive xenografts treated with 17*β*‐estradiol (E_2_). This UPR and apoptosis–paired biology underpinning estrogen-induced tumor regression not only explains the earlier observational clinical science (Haddow, 1970) but also reaffirms estrogen’s therapeutic potential for the treatment of endocrine-resistant BC.

Lønning et al. (2001) used high-dose estrogen therapy in postmenopausal women with advanced endocrine-resistant BC (median deprivation of 4 years). The conjugated equine estrogen arm in the Women’s Health Initiative trial and its long-term follow-up (Anderson *et al.*, 2004; Chlebowski *et al.*, 2020; Jordan, 2020) unintentionally illustrated the clinical relevance of estrogen-induced tumor regression (Abderrahman and Jordan, 2016). The Women’s Health Initiative trial had more than 75% of the postmenopausal women LTED for 10 years past menopause. When given estrogen replacement therapy, there were significant decreases in BC incidence and mortality (Anderson *et al.*, 2004; Roehm, 2015; Chlebowski *et al.*, 2020). Ellis et al. (2009) demonstrated the antitumor actions of low-dose estrogen therapy in postmenopausal women with advanced adjuvant aromatase inhibitor–resistant BC (deprivation ≥2 years). Iwase et al. (2013), using ethinylestradiol in patients with MBC (median age 63 years), had a 56% clinical benefit rate. Chalasani et al. (2014), using E_2_ during 3-month exemestane breaks in patients with MBC, had measurable clinical activity. These clinical studies reaffirm the earlier laboratory findings that estrogen treatment after LTED with TAM in vivo leads to BC regression (Yao et al., 2000).

These in vivo and in vitro studies and clinical trials support the clinical benefit of using estrogen alone or in combination with growth inhibitors and/or apoptosis promoters for the treatment of endocrine-resistant BC. Nonetheless, concerns regarding AEs require the development of safer estrogens.

**Fig. 1.**
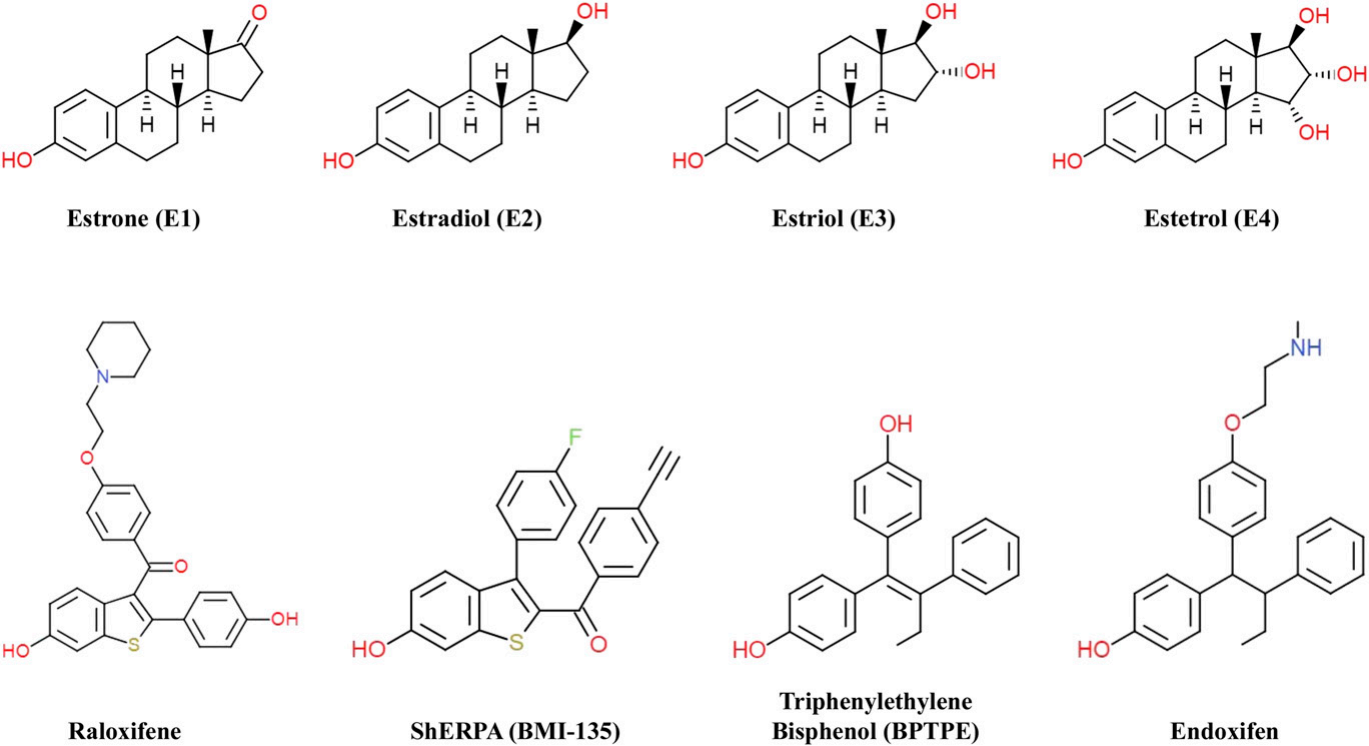
Chemical structures of naturally occurring estrogens (E_1_,E_2_, E_3_, and E_4_), SERM raloxifene, synthesized ShERPA BMI-135 based on the benzothiophene scaffold of raloxifene, partial agonist BPTPE, and SERM endoxifen. The estrogen E_2_ is the most potent estrogen. However, E_1_ is generally 12 times less potent than E_2_, and E_3_ is generally 80 times less potent than E_2_.

There are four naturally occurring forms of estrogen (Fig. 1): estrone (E_1_), E_2_, estriol (E_3_), and estetrol (E_4_).

Estetrol (Fig. 1), produced by the fetal liver during pregnancy (Holinka et al., 2008), is proposed as a promising estrogen for the treatment of advanced BC (Singer et al., 2014; Coelingh Bennink et al., 2017; Verhoeven et al., 2018; Schmidt et al., 2020), advanced prostate cancer (Dutman et al., 2017), use in hormone replacement therapy (Gérard et al., 2015; Coelingh Bennink et al., 2016; https://clinicaltrials.gov/ct2/show/NCT02834312), and in contraception (Creinin et al., 2019). In preclinical models, E_4_ selectively activates the nuclear ER*α*, which plays a prominent role in the vasculoprotective action of estrogens (Abot et al., 2014). An ongoing phase I/IIA clinical trial of E_4_ (Schmidt et al., 2020) shows the majority of patients experience favorable effects on wellbeing, and one patient completed both phases with stable disease after 24 weeks of treatment.

Selective Human ER Partial Agonists (ShERPAs), also known as selective estrogen mimics (Fig. 1), are novel benzothiophene [raloxifene (Ralox) or arzoxifene] derivatives with nanomolar potency designed to treat endocrine-resistant BC (Molloy et al., 2014; Xiong et al., 2016). The ShERPAs BMI-135 and TTC-352 were shown to cause tumor regression in TAM-resistant BC xenograft models and not to cause significant estrogen-like uterine growth in these models (Molloy et al., 2014; Xiong et al., 2016). An ongoing phase I clinical trial of TTC-352 (O’Regan et al., 2019) shows manageable safety and early clinical evidence of activity in patients with MBC progressing on endocrine therapy.

Given the clinical relevance of E_4_ and ShERPAs, here we expand the study of their pharmacology in a broad range of BC and endometrial cancer cell lines and delineate their antitumor molecular mechanisms through triggering the UPR and apoptosis in select LTED and endocrine-resistant BC models.

## Materials and Methods

### 

#### Cell Culture and Reagents.

E_1_, E_2_, E_3_, E_4_, and 4-hydroxyTAM (4OHT) were purchased from Sigma-Aldrich (St. Louis, MO). Endoxifen was purchased from Santa Cruz Biotechnology (Santa Cruz, CA), Ralox was purchased from Sigma-Aldrich, and ICI 182,780 fulvestrant (ICI) was purchased from Tocris Bioscience (Bristol, UK). Triphenylethylene bisphenol (BPTPE) was originally synthesized at the Organic Synthesis Facility, Fox Chase Cancer Center (Philadelphia, PA) (Maximov et al., 2010). The ShERPA BMI-135 was a gift from Dr. Debra Tonetti and Dr. Gregory R.J. Thatcher (University of Chicago, IL). The protein kinase regulated by RNA-like EnR kinase (PERK) inhibitor GSK G797800 was purchased from Toronto Research Chemicals (Toronto, ON, Canada). The inositol-requiring enzyme 1 (IRE1*α*) Inhibitor MKC-3946 was purchased from Calbiochem (San Diego, CA). Thioflavin T (ThT) was purchased from Sigma-Aldrich. All compounds except BMI-135 and E_4_ were dissolved in ethanol, stored at −20°C, and protected from light. Compounds BMI-135 and E_4_ were dissolved in DMSO. Wild-type (WT) estrogen-dependent BC cell line MCF-7:WS8 (Jiang et al., 1992); mutant p53 estrogen-dependent BC cell line T47D:A18 (Murphy et al., 1990b); the first in vitro cellular model recapitulating acquired-TAM resistance developed in athymic mice in vivo MCF-7:PF (Fan et al., 2014); estrogen-responsive, ER-positive, progesterone receptor–positive, and human epidermal growth factor receptor 2–positive luminal B BC cell line BT-474 (Kraus et al., 1987); estrogen-responsive, ER-positive, progesterone receptor–positive, and androgen receptor–positive luminal A BC cell line ZR-75-1 (Engel et al., 1978); antihormone-resistant estrogen-independent BC cell line MCF-7:5C (Lewis et al., 2005b); antihormone-sensitive estrogen-independent BC cell line MCF-7:2A (Pink et al., 1995); and antihormone (Ralox)-resistant estrogen-independent BC cell line MCF-7:RAL (Liu et al., 2003) were cultured as previously described. Human endometrial adenocarcinoma cell line Ishikawa was cultured as previously described (Nishida et al., 1985). All cell cultures were done in T75 and T175 culture flasks (Thermo Fisher Scientific, Waltham, MA), passaged twice a week at 1:3 ratio, and grown in 5% CO_2_ at 37°C. All cell lines were validated according to their short tandem repeat profiles at The University of Texas MD Anderson Cancer Center Characterized Cell Line Core. The short tandem repeat patterns of all cell lines were consistent with those from the Characterized Cell Line Core standard cells (Supplemental Table 1).

#### Cell Viability and Proliferation Assays.

The biologic properties of test compounds (E_1_, E_2_, E_3_, E_4_, BMI-135, BPTPE, 4OHT, endoxifen, raloxifene, and ICI) in cells lines (MCF-7:WS8, T47D:A18, MCF-7:PF, BT-474, ZR-75-1, MCF-7:5C, MCF-7:2A, and MCF-7:RAL) were evaluated by assessing the DNA content of the cells as a measure of cell viability and proliferation using a DNA fluorescence Quantitation kit (Bio-Rad Laboratories, Hercules, CA) as previously described (Fan et al., 2013). The EC_50_ of all test compounds in different human BC and human endometrial cancer cell lines are summarized in Table 1. EC_50_ was calculated using the formula: Y = Bottom + (Top-Bottom)/(1 + 10^((LogEC_50_-X)*HillSlope)).

**TABLE 1:** EC_50_ of test compounds in different human BC and human endometrial cancer cell lines The EC_50_ was calculated to indicate potency differences between test compounds used in treating these cell lines over a specific period of time (Figs. 2 and 4), as indicated in the table.

Cell Line	Time Frame	Compound	E_1_	E_2_	E_3_	E_4_	BMI-135
MCF-7:5C	1 wk	EC_50_ (−log [M])	−9.19	−10.89	−10.04	−8.73	−8.39
MCF-7:5C	2 wk		−10.00	-	−10.31	−9.30	−8.98
MCF-7:2A	2 wk		−8.99	−10.74	−9.14	−8.50	−8.29
MCF-7:RAL	1 wk		−11.11	−12.99	−9.13	−10.07	−9.97
MCF-7:RAL	2 wk		−8.93	−11.06	−9.04	−7.22	−9.24
MCF-7:RAL	3 wk		−9.53	−10.89	−9.75	−8.38	−7.47
MCF-7:PF	1 wk		−8.68	−10.67	−9.43	−8.52	−8.87
MCF-7:WS8	1 wk		−10.01	−11.92	−10.81	−9.80	−9.01
T47D:A18	1 wk		−9.33	−11.25	−10.01	−8.98	−8.87
BT-474	1 wk		-	−11.31	-	-	−8.71
ZR-75-1	1 wk		-	−11.39	-	-	−8.21
Ishikawa	1 wk		-	−10.97	-	−8.26	−8.57

#### Real-Time Polymerase Chain Reaction.

MCF-7:WS8 and MCF-7:5C cells were seeded into six-well plates at a density of 100,000 cells/well. Cells were treated the next day with test compounds (E_2_, BMI-135, BPTPE, and endoxifen) for 24 hours. RNA isolation, cDNA synthesis, and real-time polymerase chain reaction (RT-PCR) were performed as previously described (Obiorah et al., 2014). All primers were obtained from Integrated DNA Technologies Inc. (IDT, Coralville, IA) and validated by melt-curve analysis that revealed single peaks for all primer pairs. The primer sequences used for human trefoil factor 1 (TFF1) cDNA amplification were: 5′-CAT​CGA​CGT​CCC​TCC​AGA​AGA-3′ sense, 5′-CTC​TGG​GAC​TAA​TCA​CCG​TGC​TG-3′ antisense; human Growth Regulation by Estrogen in Breast Cancer 1 (GREB1) gene: 5′-CAAAGAATAACCTGTTGGCCCTGC-3′sense, 5′-GAC​ATG​CCT​GCG​CTC​TCA​TAC​TTA-3′ antisense; and the reference gene 36B4: 5′-GTG​TCC​GAC​AAT​GGC​AGC​AT-3′ sense, 5′-GAC​ACC​CTC​CAG​GAA​GCG​A-3′ antisense.

#### Transient Transfection and Dual Luciferase Reporter Assays.

Ishikawa cells were seeded into 24-well plates at a density of 100,000 cells/well. After 24 hours, cells were transfected with 28.8 μg of pERE(5X)TA-ffLuc and 9.6 μg of pTA-srLuc reporter plasmids using 3 μl of TransIT-LT1 transfection reagent (Mirus Biolabs, Madison, WI) per 1 μg of plasmid DNA in 52.5 ml of OPTI-MEM serum-free media (Invitrogen, Carlsbad, CA). Transfection mix containing the transfection complexes was added to cells in growth media to a final concentration of 0.3 μg pERE(5X)TA-ffLuc and 0.1 μg of pTA-srLuc reporter plasmids per well. After 18 hours, transfection reagents were removed, and fresh media were added instead. After 24 hours post-transfection, cells were treated with test compounds (E_2_, E_4_, BMI-135, BPTPE, and endoxifen) for 24 hours. After 24-hour treatment, cells were washed once with cold Dulbecco's phosphate-buffered saline (DPBS) (Invitrogen) and lysed, and the estrogen-responsive element (ERE) luciferase activity was determined using Dual-Luciferase Reporter Assay System (Promega, Madison, WI) according to manufacturer’s instructions. Samples were quantitated on a Synergy H1 plate reader (BioTek Instruments Inc., Winooski, VT) in white-wall 96-well plates (Nalge Nunc International, Rochester, NY).

#### Chromatin Immunoprecipitation Assays.

The chromatin immunoprecipitation (ChIP) assay was performed as previously described (Sengupta et al., 2010; Obiorah et al., 2014). The antibodies used for the pull-downs were anti-ER*α* clone F-10X mouse monoclonal (2 μg/μl; 5 μg per reaction) (Santa Cruz Biotechnology), anti–steroid receptor coactivator 3 (SRC-3) clone AX15.3 mouse monoclonal (1 μg/μl; 5 μg per reaction) (Abcam, Cambridge, UK), and normal mouse IgG as intraperitoneal negative control (2 μg/μl; 5 μg per reaction) (Santa Cruz Biotechnology). The DNA fragments were purified using Qiaquick polymerase chain reaction (PCR) purification kit (Qiagen, Germantown, MD). Then, 2 μl of eluted DNA was used for RT-PCR analysis. The primer sequences used were GREB1 proximal ERE enhancer site amplification: 5′-GTG​GCA​ACT​GGG​TCA​TTC​TGA-3′ sense and 5′-CGA​CCC​ACA​GAA​ATG​AAA​AGG-3′ antisense (Integrated DNA Technologies). The data are expressed as percent input of starting chromatin material after subtracting the percent input pull-down of the intraperitoneal negative control.

#### Docking of BMI-135 to ER*α*.

The experimental complex structure of TTC-352:ER*α* was employed for docking BMI-135:ER*α* because BMI-135 could not crystallize with the ER ligand-binding domain (LBD). The structure was prepared using Maestro software (Schrödinger Release 2019-3; Schrödinger, LLC, New York, NY, 2019) and Protein Preparation Wizard (Schrödinger Release 2019-3: Epik, Impact, Prime; Schrödinger, LLC, 2019). Briefly, the workflow involves the following steps: addition of hydrogen atoms, correction of bonds and bond order assignments, deletion of water molecules beyond 5 Å of a heteroatom, generation of ionization states at pH 7.4, and, finally, the restrained refinement of the ligand-receptor complex. The polar amino acids Asp, Glu, Arg, and Lys were modeled as charged and all Tyr were modeled as neutrals. The ligand was prepared for simulation using the LigPrep module (Schrödinger Release 2019-3; Schrödinger, LLC, 2019) in default settings. The experimental structure of ER*α* in complex with E_2_ was resolved with Tyr537 mutated to Ser. Since all biologic experiments were performed against the WT receptor, we modeled the experimental structure by mutating Ser537 to Tyr using the Maestro software. Then, the residues within a range of 5 Å of Tyr537 were refined while the remaining protein-ligand complex was kept frozen. The ligand was docked to the active site of WT ER*α* using Induced Fit Docking (Schrödinger Release 2019-3: Glide, Prime; Schrödinger, LLC, 2019) based on Prime and Glide docking (Sherman et al., 2006a,b). This methodology takes into account the receptor’s flexibility, allowing the side-chain and backbone movements in the binding site to better adjust to the shape and binding mode of the ligand. The grid was centered on the cocrystallized ligand, and the receptor van der Waals radii of the heavy atoms were scaled down to 0.5. The residues within 5 Å of ligand poses were selected to be refined. The extraprecision option was selected for docking. The top 20–ranked ligand-receptor structures were retained, and the best docking solution was selected based on the Induced Fit Docking score and visual inspection.

#### Molecular Dynamics Simulations.

Molecular dynamics (MD) simulations for the selected BMI-135:ER*α* complex were carried out with Desmond software (Schrödinger Release 2019-3, Schrödinger, LLC, 2019), utilizing the methodology previously described (Maximov et al., 2020). Briefly, the System Builder module of Desmond was used to solvate the ligand:receptor complex in a periodic orthorhombic water box based on the transferable intermolecular potential with 3 points (TIP3P) model. The charge neutrality of the system was guaranteed by adding sodium and chloride ions. To relax and equilibrate the system, Desmond’s default relaxation protocol was employed. Minimization was followed by 50-nanosecond MD production run performed in periodic boundary conditions in the isothermal–isobaric (NPT) ensemble at constant pressure and temperature of 1 atm and 300 K, respectively. The integration time step and the recording interval of coordinates were set to 2 femtoseconds and 2 picoseconds, respectively. Trajectory analysis was carried out using the analysis tool Simulation Integration Diagram of Maestro. The root-mean-square deviation (RMSD) and root-mean-square fluctuation (RMSF) of the receptor backbone atoms relative to the reference structure were calculated and compared with the same metrics computed for the trajectories of ER*α* bound to E_2_ and BPTPE, respectively [previously published (Maximov et al., 2020)]. The clustering algorithm of Desmond was used to extract the most representative frames of trajectory in terms of the conformational space sampling. The trajectory was clustered, the top 10 most-populated clusters were retained, and the representative structure of each cluster was extracted. Then, free binding energy calculations were performed with the Molecular Mechanics/Generalized Born Surface Area (MM-GBSA) method implemented in Schrödinger 2019-3 to select the best structure for analysis and comparison with the E_2_ complex. Moreover, protein-ligand interactions (e.g., H-bonds and hydrophobic contacts) were monitored throughout the simulation. All graphs were prepared using the ggplot package of R software (R, version 3.2.3; The R Foundation, Vienna, Austria, 2015), and the figures were generated using PyMol 2.0 (Schrödinger, LLC, 2019).

#### Human Unfolded Protein Response RT^2^ PCR Profiler PCR Arrays (Real-Time Profiler Assay).

MCF-7:5C cells were seeded into six-well plates at a density of 200,000 cells/well for the 48- and 72-hour time points and 45,000 cells/well for day-7 time point. After 24 hours, cells were treated with test compounds (E_2_, E_4_, BMI-135, and BPTPE). Cells were harvested using Qiazol reagent (Qiagen, Hilden, Germany), and total RNA was isolated using an miRNeasy Mini Kit (Qiagen) according to manufacturer’s instructions. During the RNA purification process, samples were treated with DNase using the RNase-Free DNase Set (Qiagen) according to manufacturer’s instructions. The cDNA was reverse-transcribed using 2 μg of isolated RNA and the High Capacity cDNA Reverse Transcription Kit (Applied Bioscience, Carlsbad, CA) according to manufacturer’s instructions. The cDNA was diluted 1:50, and a 2x RT^2^ SYBR Green Mastermix (Qiagen) was used to prepare the reactions. The plates were loaded and run on a QuantStudio 6 Flex Real-Time PCR thermocycler (Applied Bioscience) according to manufacturer’s instructions. The Ct values were exported at the end of each run, compiled, and uploaded to Qiagen’s Data Analysis Center for analysis. For the volcano plots, the fold change [2^(−*ΔΔ*CT)] in the normalized gene expression [2^(−*Δ*CT)] in the test sample divided the normalized gene expression [2^(−*Δ*CT)] in the control sample. Fold regulation represents fold-change results in a biologically meaningful way. Fold-change values greater than one indicate a positive regulation or an upregulation, and the fold regulation is equal to the fold change. Fold-change values less than one indicate a negative regulation or downregulation, and the fold regulation is the negative inverse of the fold change. The *P* values of the volcano plots were calculated using a Student’s *t* test of the replicate 2^(−*Δ*CT) values for each gene in the control group and treatment groups.

#### Live Cell Imaging and Analysis.

MCF-7:5C cells were seeded into 15 *μ*-slide two-well chambered coverslip slides (Ibidi, Martinsried, Germany) at a density of 300,000 cells/well for the 48-hour time point and at 200,000 cells/well for the 72-hour time point. After 24 hours, cells were treated with test compounds (E_2_, E_4_, BMI-135, and thapsigargin). On the day of live cell imaging, the green fluorescent dye ThT (UPR-indicative dye) (Sigma-Aldrich) was freshly prepared as previously described (Beriault and Werstuck, 2013), and the blue fluorescent live cell nuclear dye Hoechst 33342 (counterstain dye) (Thermo Fisher Scientific) was freshly prepared at a final concertation of 5 μg/ml. The staining with ThT was for 1 hour, and this was followed by substituting the culture media (containing test compounds and ThT) with PBS containing Hoechst 33342 for 15 minutes in a CO_2_ incubator. Fluorescent images of MCF-7:5C live cells were taken at a 38-millisecond exposure under a 20×/0.7 objective with ZEISS Celldiscoverer 7 (Carl Zeiss AG, Oberkochen, Germany). Images were converted to 12-bit before being quantified by the ZEISS Zen Software Module-Image Analysis. Cells from each image were manually counted to normalize the fluorescent data per cell. Relative intensity per cell = ThT intensity/cell count and was generated for each treatment per image. A mean of the relative intensity per cell (using three images per treatment) was then calculated to give a final quantification alongside the S.D. The relative intensity per cell data are represented in Table 2. The excitation and emission settings were Hoechst 33342 (Excitation: 348 nm, Emission: 455 nm) and ThT (Excitation: 433 nm, Emission: 475 nm).

**TABLE 2:** Quantification of the UPR in live MCF-7:5C cells through measuring ThT relative intensity/cell (A) ThT relative intensity/cell (mean and S.D.) with test compounds after 48-hour treatments. (B) ThT relative intensity/cell (mean and S.D.s) after 72-hour treatments. This reflects the differential capacity of test compounds in inducing EnR stress over time. ThT relative intensity/cell per treatment is representative of three biologic repeats.

	(A) Day 2	
Compound	Relative Intensity/Cell (Mean)	S.D.
Veh	0.276	0.052
Thapsigargin	0.875	0.061
E_4_	1.245	0.073
E_2_	0.741	0.097
BMI-135	0.497	0.047
	(B) Day 3	
Compound	Relative Intensity/Cell (Mean)	S.D.
Veh	0.296	0.057
Thapsigargin	10.055	0.068
BMI-135	4.878	0.049

#### Annexin V–Staining Assays.

MCF-7:5C cells were seeded into 10-cm Petri dishes at a density of 800,000 cells/dish for the 72- and 96-hour time points. MCF-7:2A cells were seeded into 10-cm Petri dishes at a density of 400,000 cells/dish for day-9 time point and at 100,000 cells/dish for day-13 time point. MCF-7:RAL cells were seeded into 10-cm Petri dishes at a density of 150,000 cells/dish for day-14, day-17, and day-21 time points. After 24 hours, cells were treated with test compounds (E_2_, E_4_, BMI-135, BPTPE, 4OHT, endoxifen, raloxifene, ICI, GSK G797800, and MKC-3946). Harvested cells were suspended in 1× binding buffer, and 1 × 10^5^ cells were stained simultaneously with FITC-labeled annexin V and propidium iodide (PI) for 15 minutes at 37°C using the FITC Annexin V Apoptosis Detection Kit I (BD Pharmingen, San Diego, CA) according to the manufacturer’s instructions. The cells were analyzed using a BD Accuri C6 plus flow cytometer.

#### Statistical Analyses.

All data are mean ± S.D. of three different fields for each condition from three independent biologic experiments performed in technical duplicates. One-way ANOVA was used with a follow-up Tukey’s test to determine the statistical significance of the treatments.

## Results

### 

#### Effects of E_4_ and BMI-135 on Cell Viability and Proliferation in Numerous BC Models.

Cell viability and proliferation assays were used to investigate the biologic properties of test compounds. Estetrol and ShERPA BMI-135 display activity similar to E_2_ but right shifted across eight BC cell lines that are estrogen-dependent (MCF-7:WS8, T47D:A18, MCF-7:PF, BT-474, and ZR-75-1), estrogen-independent (MCF-7:5C, MCF-7:2A, and MCF-7:RAL), endocrine-sensitive (MCF-7:2A), endocrine-resistant (MCF-7:PF, MCF-7:5C, and MCF-7:RAL), mutant p53 (T47D:A18), human epidermal growth factor receptor 2–positive (BT-474), luminal A (ZR-75-1), and luminal B (BT-474).

**Fig. 2.**
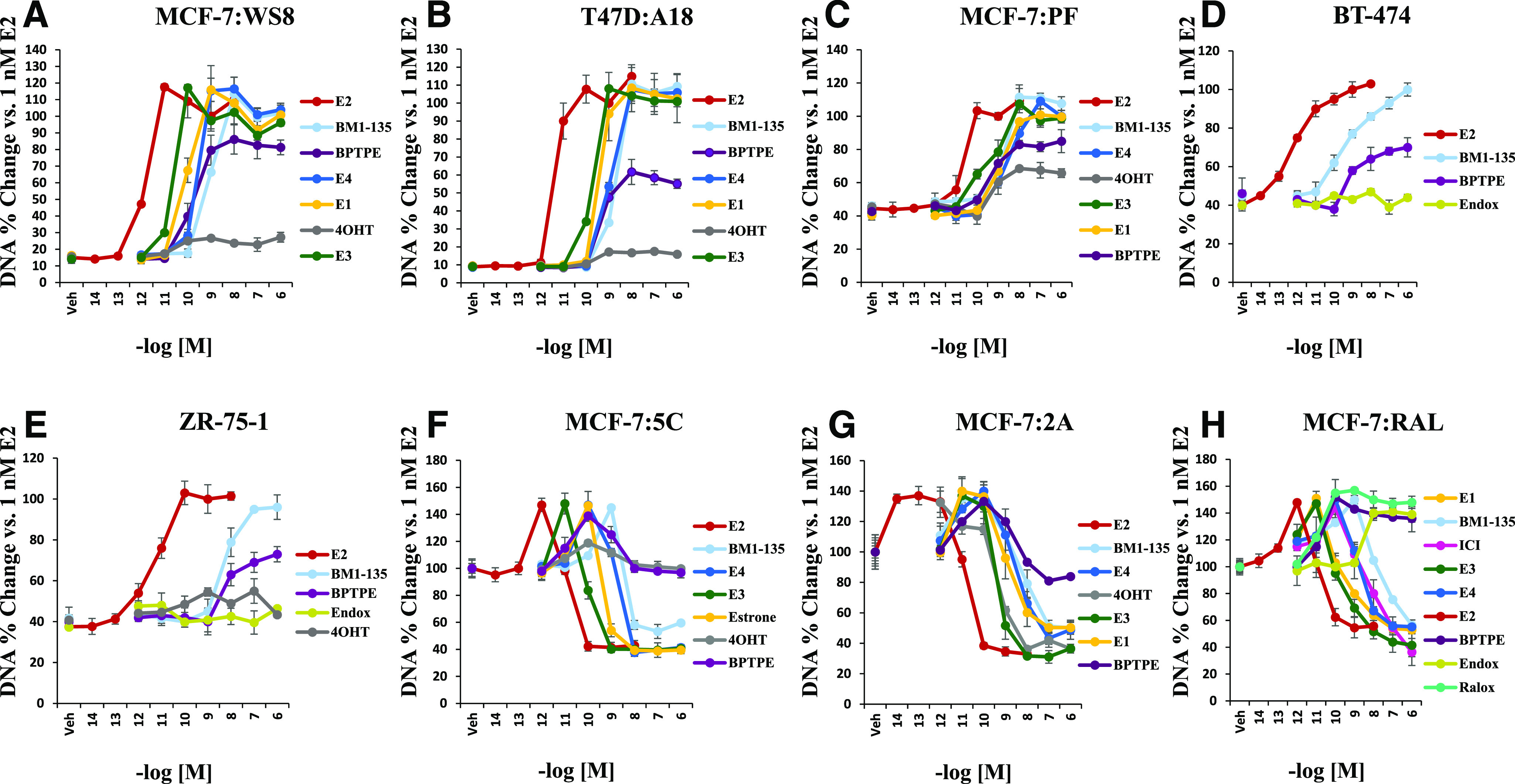
Cell viability and proliferation assays in multiple BC cell lines with test compounds. (A) Effects of test compounds alone after 7 days of treatment (percent DNA of vehicle vs. test compounds’ concertation) in MCF-7:WS8. (B) Effects of test compounds alone after 7 days of treatment in T47D:A18. (C) Effects of test compounds alone after 7 days of treatment in MCF-7:PF. (D) Effects of test compounds alone after 7 days of treatment in BT-474. (E) Effects of test compounds alone after 7 days of treatment in ZR-75-1. (F) Effects of test compounds alone after 7 days of treatment in MCF-7:5C. (G) Effects of test compounds alone after 14 days of treatment in MCF-7:2A. (H) Effects of test compounds alone after 21 days of treatment in MCF-7:RAL. Endox, endoxifen.

The concentration 1 μM for E_4_ and BMI-135 achieved either the maximal cellular growth (Fig. 2, A–E; Supplemental Fig. 1, A–C), or the maximal cellular death (Fig. 2, F–H; Supplemental Fig. 1, D–F). Both were shown to be less potent full agonists compared with E_2_, requiring higher concentrations to produce the same maximal effect of E_2_. The EC_50_ for all test compounds used in treating these cell lines are summarized in Table 1.

In MCF-7:5C, E_4_ and BMI-135 almost completely reduced the amount of viable MCF-7:5C cells after 1 week of treatment in a dose-dependent manner, with a maximum reduction of cells by an average of 58% for E_4_ and 46% for BMI-135 at their highest concentration of 10^−6^ M (*P* < 0.05 compared with vehicle) (Fig. 2F). Reduction in the amount of viable MCF-7:5C cells by E_2_ at 10^−9^ M was by an average of 58% (Fig. 2F). In MCF-7:2A, E_4_ and BMI-135 almost completely reduced the amount of viable MCF-7:2A cells after a 2-week treatment in a dose-dependent manner, with a maximum reduction of cells by an average of 57% for E_4_ and 50% for BMI-135 at their highest concentration of 10^−6^ M (*P* < 0.05 compared with vehicle) (Fig. 2G). Reduction in the amount of viable MCF-7:2A cells by E_2_ at 10^−9^ M was by an average of 67% (Fig. 2G). In MCF-7:RAL, E_4_ and BMI-135 almost completely reduced the amount of viable MCF-7:RAL cells after a 3-week treatment in a dose-dependent manner, with a maximum reduction of cells by an average of 45% for E_4_ and 43% for BMI-135 at their highest concentration of 10^−6^ M (*P* < 0.05 compared with vehicle) (Fig. 2H). Reduction in the amount of viable MCF-7:RAL cells by E_2_ at 10^−9^ M was by an average of 45% (Fig. 2H).

#### Effects of E_4_ and BMI-135 Are Mediated via ER*α*.

MCF-7:5C, MCF-7:2A, and MCF-7:RAL representing LTED estrogen-independent BC were treated with 1 μM E_4_, 1 μM BMI-135, and a combination of these with 1 μM 4OHT and 1 μM endoxifen to investigate whether E_4_ and BMI-135 exert their function via ER*α*. In MCF-7:5C, full estrogen agonists should cause cellular death within 1 week, antagonists should not (i.e., MCF-7:5C is endocrine-resistant), and the agonists’ pairing with the antagonists should block the death effect. Indeed, E_2_, E_4_, and BMI-135 killed the cells within 1 week (*P* < 0.05 compared with vehicle) (Supplemental Fig. 2A), whereas 4OHT and endoxifen did not (*P* < 0.05 compared with vehicle) (Supplemental Fig. 2A). The combination of E_2_, E_4_, and BMI-135 with 4OHT and endoxifen blocked the death effect (Supplemental Fig. 2A).

In MCF-7:2A, full agonists should cause cellular death within 2 weeks, antagonists should cause growth inhibition (i.e., MCF-7:2A is endocrine-sensitive), and the agonists’ pairing with the antagonists should block the death effect. Indeed, E_2_, E_4_, and BMI-135 killed the cells within 2 weeks (*P* < 0.05 compared with vehicle) (Supplemental Fig. 2B), whereas 4OHT and endoxifen caused growth inhibition (*P* < 0.05 compared with vehicle) (Supplemental Fig. 2B). The combination of E_2_, E_4_, and BMI-135 with 4OHT and endoxifen blocked the death effect (Supplemental Fig. 2B).

In MCF-7:RAL cells, full agonists should cause cellular death within 2 to 3 weeks in vitro; antagonists, especially Selective ER Modulator (SERM) raloxifene (positive control), should cause cellular growth; and the agonists’ pairing with antagonists should block the death effect. Indeed, E_2_, E_4_, and BMI-135 killed the cells within 3 weeks (*P* < 0.05 compared with vehicle) (Supplemental Fig. 2C), whereas the SERMs 4OHT, endoxifen, and especially raloxifene caused cellular growth (*P* < 0.05 compared with vehicle) (Supplemental Fig. 2C). The combination of E_2_, E_4_, and BMI-135 with 4OHT and endoxifen blocked the death effect (Supplemental Fig. 2C). Interestingly, ICI (a selective ER downregulator or “pure antiestrogen”) caused a decrease in cell DNA amount in MCF-7:RAL cells after a 3-week treatment (*P* < 0.05 compared with vehicle) (Supplemental Figs. 1F and 2C).

Endoxifen, the major biologically active metabolite of TAM, was used as an antiestrogenic control alongside 4OHT and neither induced an increase or decrease in viable cells (*P* < 0.05 compared with vehicle controls) (Supplemental Fig. 2A). Only in MCF-7:2A cells did 4OHT and endoxifen cause growth inhibition (Supplemental Fig. 2B), and in MCF-7:RAL cells, both caused growth stimulation (Supplemental Fig. 2C), as predicted.

#### BMI-135 Induces the Transcriptional Activity of ER*α* Similar to E_2_ in WT MCF-7:WS8 and Apoptotic-Type MCF-7:5C BC Models.

**Fig. 3.**
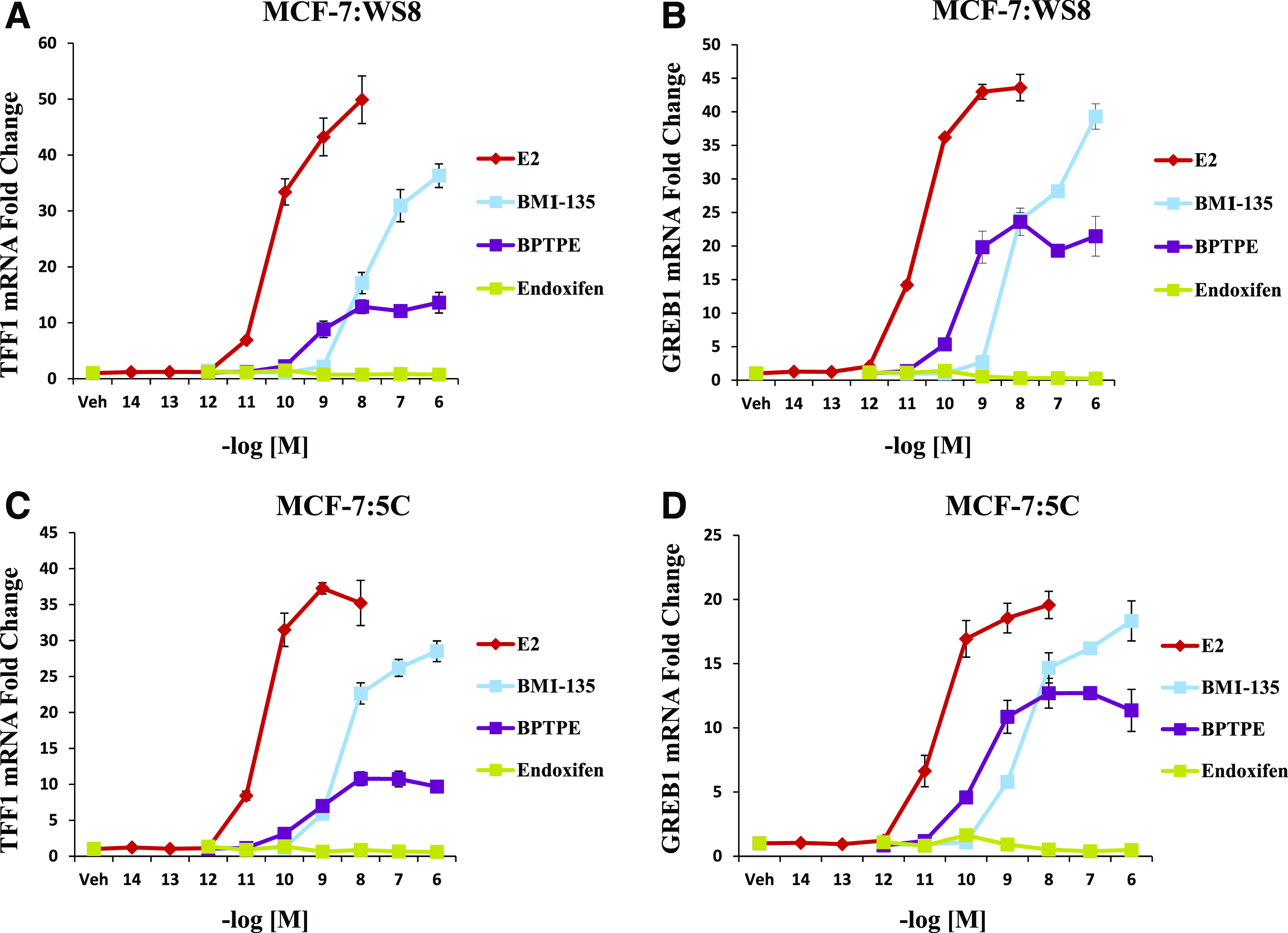
Transcriptional activity of well characterized estrogen-responsive genes TFF1 (or pS2) and GREB1 in WT MCF-7:WS8 and LTED MCF-7:5C with test compounds. (A) mRNA expression of TFF1 in MCF-7:WS8 cells after 24-hour treatment with 1 nM E_2_ and 1 µM for other test compounds. (B) mRNA expression of GREB1 in MCF-7:WS8 cells after 24-hour treatment with 1 nM E_2_ and 1 µM for other test compounds. (C) mRNA expression of TFF1 in MCF-7:5C cells after 24-hour treatment with 1 nM E_2_ and 1 µM for other test compounds. (D) mRNA expression of GREB1 in MCF-7:5C cells after 24-hour treatment with 1 nM E_2_ and 1 µM for other test compounds. Data are mean ± S.D. from three independent experiments performed in triplicate analyzed by one-way ANOVA.

Quantitative RT-PCR was used to assess the transcriptional activity of ER*α* on ERE genes (*TFF1* and *GREB1*) with test compounds. After 24-hour treatment in MCF-7:WS8 cells, BMI-135 increased the levels of *TFF1* and *GREB1* mRNAs compared with vehicle controls (*P* < 0.05) (Fig. 3, A and B). On the other hand, the partial agonist BPTPE induced a partial increase in the levels of *TFF1* and *GREB1* mRNAs and less than that of full agonist E_2_ (*P* < 0.05) and BMI-135 (*P* < 0.05) (Fig. 3, A and B). The minimal concentration that produced a complete increase in the levels of *TFF1* and *GREB1* was at 10^−6^ M for BMI-135 (*P* < 0.05 compared with vehicle) (Fig. 3, A and B).

After 24-hour treatment in MCF-7:5C cells, BMI-135 increased the levels of *TFF1* and *GREB1* mRNAs compared with vehicle controls (*P* < 0.05) (Fig. 3, C and D). On the other hand, BPTPE induced a partial increase in the levels of *TFF1* and *GREB1* mRNAs and less than that of E_2_ (*P* < 0.05) and BMI-135 (*P* < 0.05) (Fig. 3, C and D). The minimal concentration that produced a complete increase in the levels of *TFF1* and *GREB1* was at 10^−6^ M for BMI-135 (*P* < 0.05 compared with vehicle) (Fig. 3, C and D).

The ERE-dependent transcriptional activity with E_4_ was done by Abot et al. (2014) and showed an induction similar to E_2_, only with a lower potency.

Overall, the induction of the mRNA levels of *TFF1* and *GREB1* by BMI-135 in MCF-7:WS8 and MCF-7:5C was similar to that by full agonist E_2_, only at a lower potency.

#### Estetrol and BMI-135 Induce the Transcriptional Activity of ER*α* Similar to E_2_ in Human Endometrial Cancer Model Ishikawa.

**Fig. 4.**
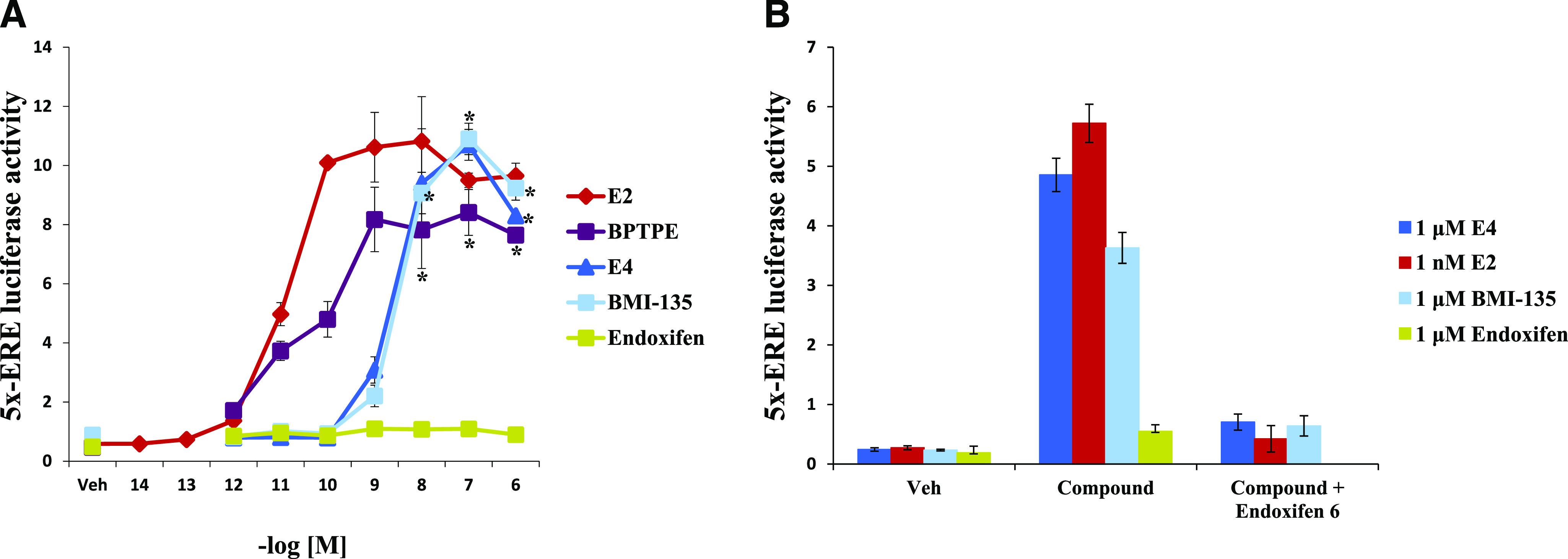
Transient transfection of the human endometrial cancer cells Ishiskawa with 5x-ERE and dual luciferase activity assay. (A) Dose-response curve of test compounds vs. 5x-ERE luciferase activity (promoter activity = Firefly luciferase activity/*Renilla* luciferase activity). (B) 5x-ERE luciferase activity with indicated test compounds alone vs. in combination with 1 μM endoxifen. Data are mean ± S.D. from three independent experiments performed in triplicate analyzed by one-way ANOVA. *P < 0.05: statistical difference between E_4_ or BMI-135 and BPTPE treatments over 10^−8^–10^−6^ concentration range (*t* test). Veh, vehicle.

Transient transfection and luciferase activity assays were used to determine the transcriptional activity of ER*α* on estrogen-responsive genes (*5xERE*) with test compounds as ERE dual luciferase activity. After 24-hour treatment of Ishikawa cells, E_4_ and BMI-135 increased the levels of 5x-ERE luciferase activity compared with vehicle controls (*P* < 0.05) (Fig. 4A). On the other hand, the partial agonist BPTPE induced a partial increase in the levels of 5x-ERE luciferase activity and less than that of full agonist E_2_, E_4_, and BMI-135 (*P* < 0.05) at concentration range of 10^−8^–10^−6^ M (Fig. 4A). The minimal concentration that produced a complete increase in the levels of 5x-ERE luciferase activity was at 10^−7^ M for E_4_ and BMI-135 (*P* < 0.05 compared with vehicle) (Fig. 4A).

To determine whether the effects of E_4_ and BMI-135 were mediated via ER*α* in Ishikawa cells, transiently transfected Ishikawa cells were treated with test compounds in combination with antagonist endoxifen for 24 hours, and luciferase activity assays were conducted (Fig. 4B). The increase in the levels of 5x-ERE luciferase activity with E_4_ and BMI-135 was blocked with endoxifen treatment at 10^−6^ M (*P* < 0.05 compared with vehicle) (Fig. 4B). This confirms that E_4_ and BMI-135 exert their function via Ishikawa’s ER*α*. In addition, endoxifen alone did not increase the levels of 5x-ERE luciferase activity in Ishikawa cells, acting as an antagonist in this uterine model (Fig. 4B).

Overall, the induction of the levels of 5x-ERE luciferase activity by E_4_ and BMI-135 in Ishikawa cells was similar to that by full agonist E_2_, only at a lower potency (Table 1).

#### E_4_ and BMI-135 Recruit ER*α* and SRC-3 to the *GREB1* Proximal Enhancer Region Similar to E_2_ in MCF-7:5C BC Model.

**Fig. 5.**
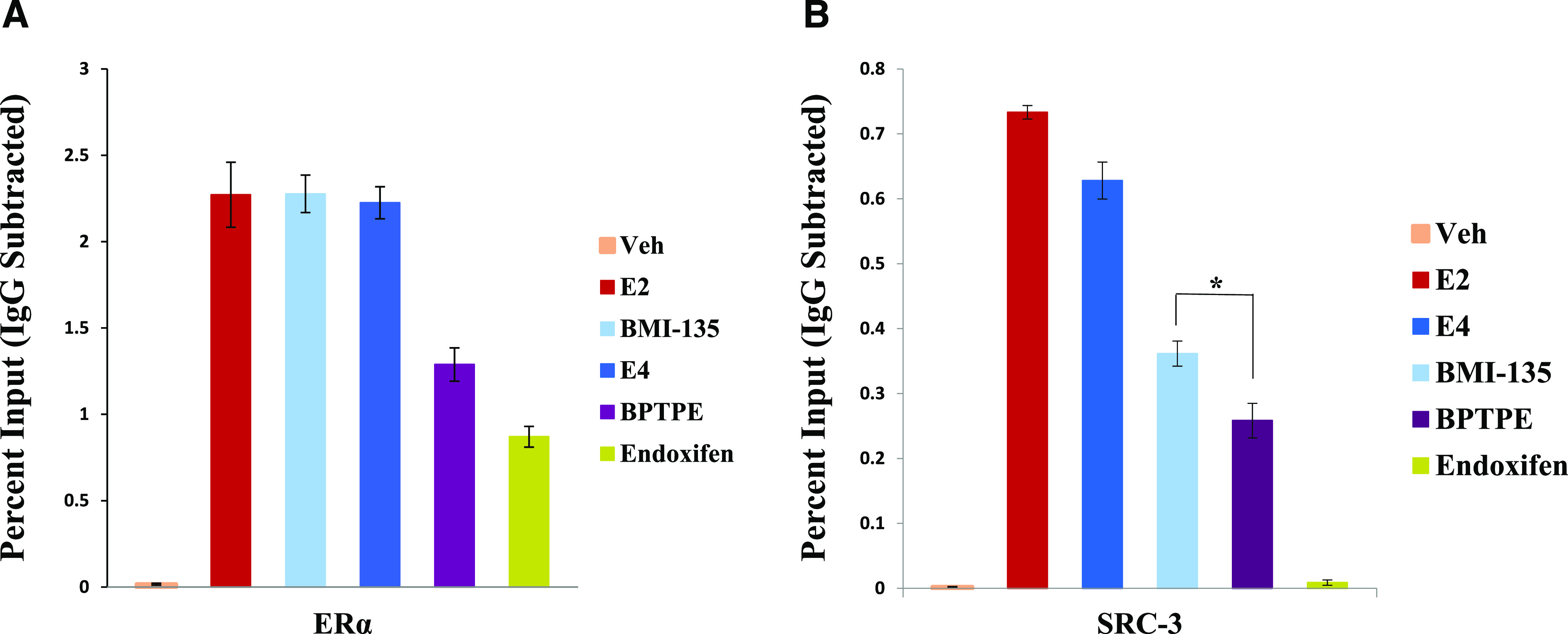
ChIP assay in MCF-7:5C cells showing the recruitment of ER*α* and coactivator SRC-3 to TFF1 ERE promoter. Recruitment of ER*α* (A) and SRC-3 (B) after 45-minute treatment with indicated ligands; 1 nM E_2_ and 1 μM for the rest of test compounds. Recruitment of ER*α* and SRC-3 was calculated as percentage of the total input after subtracting the IgG recruitment. All treatments were performed in triplicate; data represent the average of these replicates. *P < 0.05: statistical difference between BMI-135 and BPTPE treatments with SRC-3 recruitment. Veh, vehicle.

ChIP assays were used to assess the recruitment of ER*α* and SRC-3 to the *GREB1* proximal enhancer region with test compounds. Estetrol and BMI-135 treatments resulted in a very strong recruitment of ER*α* to the *GREB1* proximal enhancer region similar to E_2_ and higher than that with the partial agonist BPTPE (*P* < 0.05) (Fig. 5A).

However, the recruitments of the coactivator SRC-3 to the *GREB1* proximal enhancer region with E_4_ and BMI-135 treatments were higher than that with BPTPE (*P* < 0.05) (Fig. 5B). SRC-3 recruitment with E_2_ was the highest. With E_4_, there was an 18.72% recruitment reduction compared with E_2_; with BMI-135, there was a 51.17% recruitment reduction compared with E_2_; with BPTPE, there was a 65.47% recruitment reduction compared with E_2_; and with endoxifen, there was a 98.14% recruitment reduction compared with E_2_ (Fig. 5B).

Overall, the recruitment of ER*α* to the *GREB1* proximal enhancer region with E_4_ and BMI-135 in MCF-7:5C cells was similar to that by full agonist E_2_, and the recruitment of SRC-3 to the *GREB1* proximal enhancer region with E_4_ and BMI-135 in MCF-7:5C cells was higher than that with the partial agonist BPTPE. Although SRC-3 recruitment with BMI-135 treatment was lower than that with E_2_ (*P* < 0.05), it was higher than that with BPTPE (*P* < 0.05).

#### Analysis of E_4_ and BMI-135’s Binding Mode in Comparison with Full Agonist E_2_ and Partial Agonist BPTPE.

**Fig. 6.**
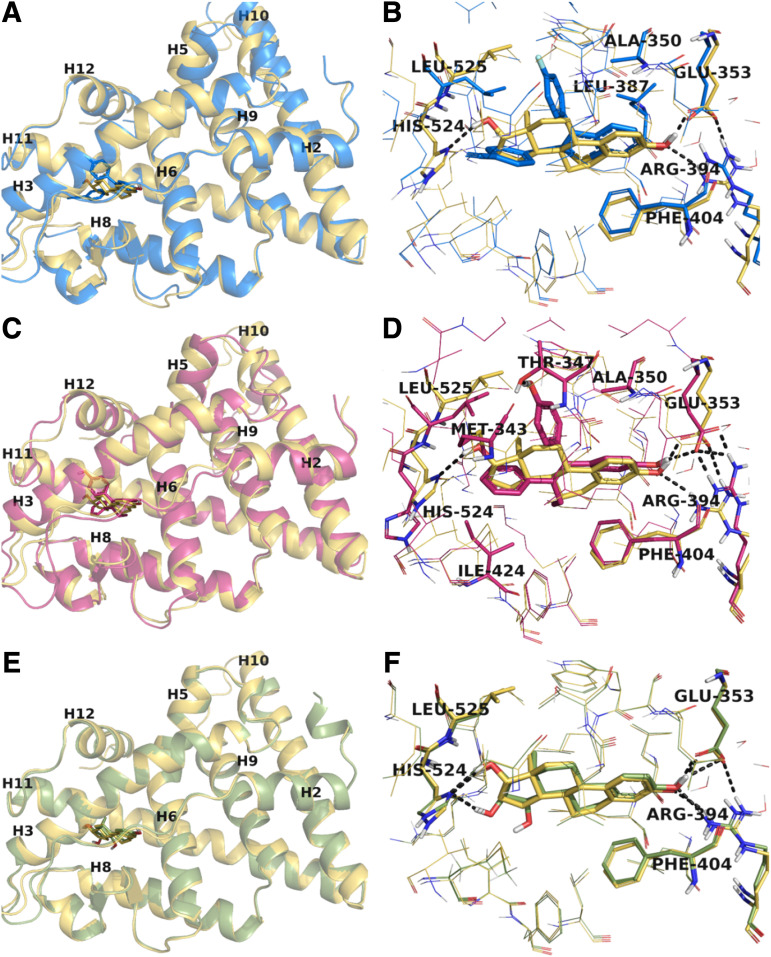
Representations of ER*α*-LBD with E_2_, E_4_, BMI-135, and BPTPE. Comparison between the agonist conformation of ER*α*-LBD in complex with E_2_, superimposed with BMI-135 (A), BPTPE (C), and E_4_ (E) in similar conformations of the receptor. The helices forming the ligand-binding site are labeled together with helix 12 (H12), which defines the receptor conformation and those essential for the coactivator binding groove. The alignment in the binding site and the contacts between BMI-135 (B), BPTPE (D), E_4_ (F), and critical amino acids of the binding pocket are revealed in comparison with the binding alignment of E_2_. For BMI-135 and BPTPE, the most representative conformations extracted from MD trajectories are shown, whereas for E_2_ and E_4_, the experimental structures are presented. The ligand:receptor complexes are colored based on C atoms as follows: yellow for E_2_, blue for BMI-135, magenta for BPTPE, and light green for E_4_, whereas the N, O, and S atoms are colored in dark blue, red, and yellow, respectively. For clarity, the amino acids involved in critical contacts (i.e., H-bonds and *π*- *π* stacking) are shown as sticks together with those having contacts with occurrence frequencies during the MD trajectories larger than 40% of the simulation time. The remaining amino acids of the binding sites are shown as lines. The H-bonds redepicted as black dashed lines.

To outline the similarities and differences between BMI-135 and other investigated ligands (e.g., E_2_, E_4_, and BPTPE), their overall conformations and interactions with residues of the binding site were analyzed (Fig. 6; Supplemental Fig. 10, B–I). The BMI-135 ligand was docked into the experimental structure of the ER*α*:TTC-352 complex and adopted the canonical agonist conformation with helix 12 (H12) positioned over the binding pocket, sealing the ligand inside. We used the induced fit docking methodology because it allows flexibility for certain parts of the receptor (e.g., amino acids of the binding site). The top-ranked BMI-135–receptor pose and experimental structures of ER*α* bound to E_2_, E_4_, and BPTPE adopt the agonist conformation of ER*α*, with H12 sitting in a groove between H5 and H11 delineated by H3 and the ligands occupying the binding pocket composed of residues from helices H3, H6, H8, and H11 (Fig. 6, A, C, and E).

The predicted binding mode of BMI-135 shared, to some extent, the network of interactions specific to E_2_, E_4_, and BPTPE, as shown (Fig. 6, B, D, and F; Supplemental Fig. 10, F–I). The familiar H-bond network between a phenolic hydroxyl, Glu353, and Arg394 was common to ligands. The benzothiophene moiety of BMI-135 was implicated in *π*-*π* stacking interactions with Phe404 and made several additional contacts with Ala350 (H3), Leu387, Met388, and Leu391 (H6), similar to A and B rings of E_2_. The two substituted phenyl rings were involved in hydrophobic contacts with Leu346 (H3), Ala350 (H3), Ile424 (H8), and Leu525 (H11), and the fluorine substituent was headed toward Thr347 (H3). The most apparent difference between BMI-135 and E_2_ binding modes (also seen for BPTPE) was the absence of H-bond with the imidazole ring of His524. We noticed that the side chain of His524 was pushed toward the outer part of the protein by the bulkier ethinyl group of BMI-135, which hovered between helices H3, H8, and H11 in a space delineated by residues Met343 (H3), Val418 (H8), Met421 (H8), Leu525 (H11), and Met528 (H11) (Supplemental Fig. 10H). These flexible residues permitted the accommodation of the large etinylbenzoyl moiety in this part of the binding pocket.

A contact unique to BPTPE was the H-bond between the second phenolic group of the ligand and the OH group of Thr347 (Fig. 6D), whereas specific to E_4_ was the involvement of the second OH group of the D ring into an extra H-bond to His524, adding stability to the ligand in the binding site (Fig. 6F). In addition, the hydrophobic contacts and *π*-*π* stacking interactions with Phe404 complemented the binding profile of these ligands (Supplemental Fig. 10, C, E, G, and I).

#### MD Simulations Analysis.

To investigate the stability of BMI-135 in the binding site of ER*α*, the dynamics of the interactions, and how they compared with the interactions in the structures of E_2_ and BPTPE, we performed MD simulations against the top-ranked ER*α*:BMI-135 complex, as previously described in *Materials and Methods*. The recorded trajectory was analyzed and compared with the trajectories previously reported (Maximov et al., 2020) for WT ER*α* bound to E_2_ and BPTPE.

Firstly, we explored the conformational stability of the simulation. To ensure that the model had reached equilibrium, RMSDs of the protein backbone atoms, relative to their position in the first frame, were computed for trajectory. The RMSD evolution indicated that the system had reached equilibrium after approximately 5 nanoseconds, similar to the E_2_ model (Supplemental Fig. 3A).

Next, to investigate the mobility of the protein and the dynamics of ligand binding, we monitored the RMSF of the residues along the trajectory (Supplemental Fig. 3A). Comparing the RMSF calculated for backbone atoms with the previously reported values for the runs of E_2_ and BPTPE, we noticed a similar pattern for BMI-135 and E_2_. There were several substantial fluctuations, which mainly overlapped with the flexible domains of the receptor (a significant peak located between residues 332–338 matches the loop connecting helices H2 and H3). The largest peak in all trajectories was situated between residues 456 and 469, part of the loop connecting H9 to H10, and missing in all experimental structures used in this analysis (Supplemental Fig. 3A). The high flexibility of this domain and the predicted coordinates for this loop could explain the observed fluctuation. Overall, the BMI-135 complex showed mobility domains matching with the E_2_ system mainly positioned in connection loops, flexible regions of a protein. In addition, based on the previous analysis of the correlation between RMSF values and B-factors for E_2_ and BPTPE, we observed that the high RMSF values of protein fragments parallel with large B-factors.

Then, we explored the stability of the ligands relative to the protein and the binding site together with the internal fluctuations of ligands’ atoms (Supplemental Fig. 3B). The analysis shows that BMI-135 did not fluctuate significantly and was stably bound in the active site, similar to E_2_ and BPTPE, with average RMSD values of 0.8 ± 0.23 and 1.6 ± 0.34 Å, respectively (Supplemental Fig. 3B).

#### Analysis of BMI-135 Ligand-Protein Interactions in Modeled WT ER*α* Systems.

We analyzed the binding dynamics of BMI-135 and assessed the stability of the interactions by monitoring the frequency of occurrence of that specific interaction throughout the trajectory. Overall, the computed variations of RMSF, based on the backbone and side-chain atoms, showed similar trends for E_2_, BMI-135, and BPTPE (Supplemental Fig. 10A). The residues involved in H-bonds with the ligands (e.g., Thr347, Glu353, His524), *π*-*π* stacking, and hydrophobic contacts (e.g., Phe404, Ala350, Leu387) showed RMSF values that were smaller than average and fluctuated less, indicating stable contacts. This observation was also supported by the occurrence frequencies of these interactions monitored throughout the trajectory (Supplemental Fig. 11, A–C). A striking difference was noticed for BMI-135, which displayed the largest peak of side-chain RMSF for Arg394. This mobility indicated that Arg394 was not involved in a direct H-bond with the ligand and/or ionic bridges to Glu353, therefore not stabilizing it. However, H-bonds were sporadically monitored during the simulation between the ligand and Arg394 via a water bridge, with frequencies below 15%. Additionally, the bulkier substituents of BMI-135 displaced the amino acid and forced it not to adopt orientations proper for the binding.

Similarly to E_2_, BMI-135 was stabilized by the H-bond to Glu353 and *π*-*π* stacking interactions with Phe404 but occurred in lower frequency. The hydrophobic contacts, mainly with residues Ala 350, Leu384, Leu 387, Met388, Leu391, Leu403, and Leu525, were stable for both ligands during the simulation time, however, in lower occurrence frequencies for BMI-135 (Supplemental Fig. 11, A and B). The H-bond to His524, which was very stable for E_2_, was lacking for BMI-135 and BPTPE, but occasional hydrophobic contacts with the ethinyl-benzoyl moiety of BMI-135 were noticed. BPTPE mainly recapitulated the interactions mentioned above but with frequencies lower than those of E_2_.

A distinctive feature of BPTPE is the H-bonding to Thr347, which occurred in over 95% of the trajectory (Supplemental Fig. 11C), indicating a very stable contact, and this was confirmed by the low RMSF value of the residue (Supplemental Fig. 10A). However, as previously shown, the H-bond to Thr347 prevented the formation of an H-bond between the side chains of Asn348 (H3) and Tyr537 (H11) (usually forming a stabilizing contact in the vicinity of H12) and, together with the phenol group of BPTPE, triggered a slightly different conformation of H12 (Maximov et al., 2020). Although the 4-fluoro-phenyl substituent of BMI-135 was oriented toward Thr347, the interaction Asn348-Tyr537 was not disturbed and occurred 52% of the simulation time but to a slightly lesser extent compared with E_2_ (i.e., 70%); nonetheless, it is still significant. Another contact that added stability to the agonist conformation of the receptor was the interaction between the side chain of His524 and backbone of Glu419, which was found almost 80% of the time during the simulation of E_2_. Surprisingly, this contact was observed in the trajectory of BMI-135 with a frequency of 72% of the simulation time.

Overall, these data show the confirmation of the BMI-135:ER*α* complex to be more similar to that of E_2_, compared with that of BPTPE.

#### E_4_ and BMI-135 Activate the UPR.

**Fig. 7.**
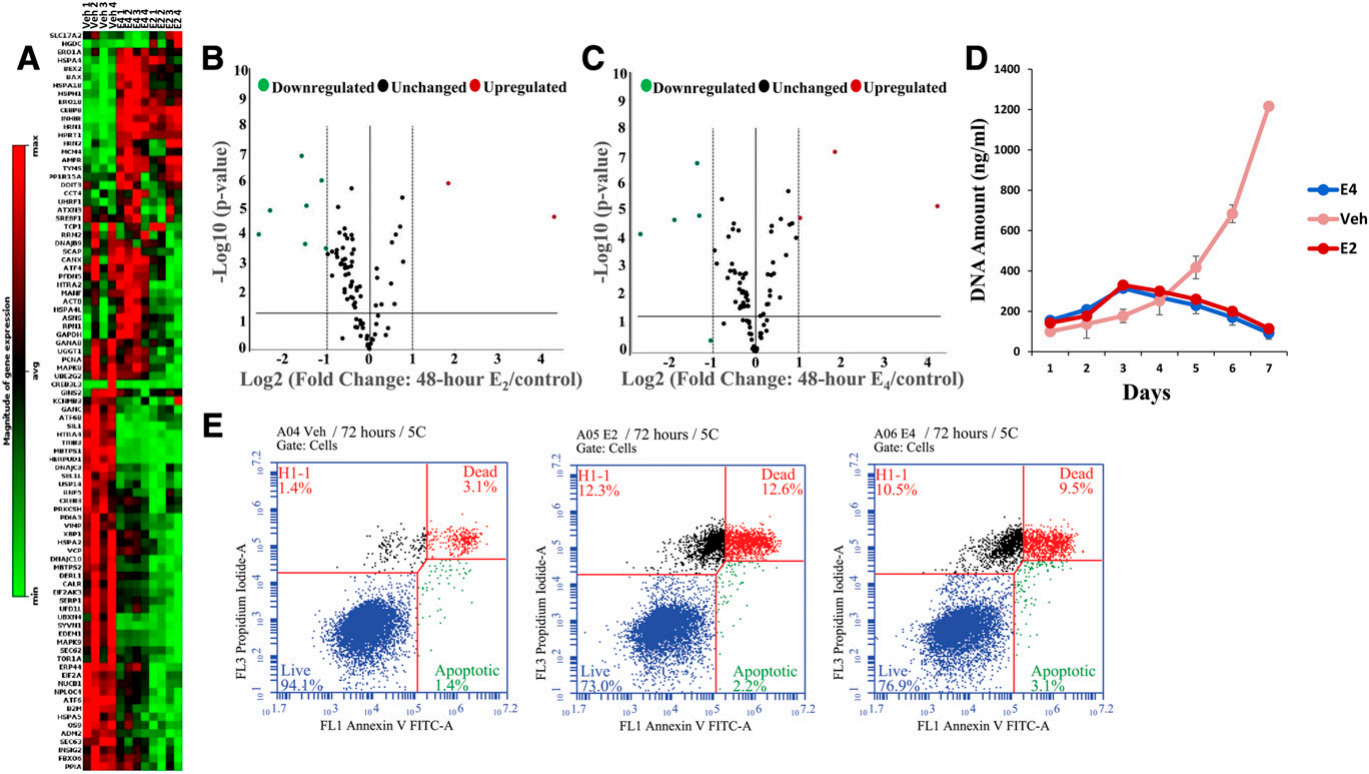
Human UPR RT^2^ PCR profiler PCR arrays, proliferation assays, and annexin V staining in MCF-7:5C cells with 48-hour, 96-hour, and 7-day E_2_ and E_4_ treatments. (A) A heat map providing a visualization of the fold changes in expression between select groups (from left to right; vehicle, E_4_, and E_2_, respectively) for every gene in the array in the context of the array layout. (B) A volcano plot of 48-hour E_2_ treatment identifying significant gene-expression changes and displaying statistical significance vs. fold change on the *y*- and *x*-axes, respectively. The volcano plot combines a *P*-value statistical test with the fold-regulation change-enabling identification of genes with both large and small expression changes that are statistically significant. (C) A volcano plot of 48-hour E_4_ treatment. (D) Effects of E_2_ and E_4_ alone after 7 days of treatment. (E) Flow cytometry of 72-hour E_2_ and E_4_ treatments. (B and C) Green represents downregulated, black unchanged, and red upregulated. Data are mean ± S.D. from three independent experiments performed in triplicate analyzed by one-way ANOVA. Veh, vehicle.

Human UPR real-time profiler assays were used to assess the regulation of UPR genes with test compounds. Cell viability and proliferation assays showed a decline in MCF-7:5C cell DNA amount with E_2_ and E_4_ treatments at 72 hours (Fig. 7D). Furthermore, flow cytometry showed apoptosis at 72 hours (annexin staining 14.8% with E_2_ and 12.6% with E_4_ vs. vehicle control 4.5%) (Fig. 7E). The time point at 48 hours was chosen to investigate the terminal (or proapoptotic) UPR gene regulation with E_2_ and E_4_ treatments in MCF-7:5C cells, which precedes apoptosis by 72 hours.

After 48-hour treatment with 1 nM E_2_ and 1 μM E_4_ [i.e., these concentrations were shown earlier to trigger maximal cellular death (Fig. 2; Table 1)], the endoplasmic reticulum–associated degradation (ERAD) genes (downstream IRE1*α*/XBP1s and ATF6 p50), HTRA4 (*P* < 0.001), SYVN1 (*P* < 0.001), and HERPUD1 (*P* < 0.001), were downregulated (Fig. 7, B and C; Supplemental Fig. 5, A and B). The lipid or cholesterol metabolism genes (downstream IRE1*α*/XBP1s and ATF6 p50), MBTPS1 (*P* < 0.001) and SERP1 (*P* < 0.001), were downregulated with E_2_ treatment, whereas only MBTPS1 (*P* < 0.001) was downregulated with E_4_ (Fig. 7, B and C; Supplemental Fig. 5, A and B). The chaperone (chaperones are usually downstream IRE1*α*/XBP1s, PERK/P-eIF2*α*:ATF4, and ATF6 p50) gene SIL1 (*P* < 0.001) was downregulated with E_4_ treatment (Fig. 7C; Supplemental Fig. 5B). By contrast, the genes CEBPB (*P* < 0.001) and INHBE (*P* < 0.001), which reflect high UPR stress, were upregulated (Fig. 7, B and C; Supplemental Fig. 5, A and B).

The heat map of MCF-7:5C cells with E_2_ and E_4_ treatments at 48 hours displays a general UPR gene downregulation (situated on the right side of the heat map) compared with vehicle control (situated on the left) (Fig. 7A). The majority of the profiler assays’ genes belong to the lipid metabolism, ERAD, and chaperone gene groups, which are considered prosurvival mechanisms that help the cells cope with extrinsic or intrinsic cellular stress (Fig. 9). This general downregulation by 48 hours (Fig. 7, B and C; Supplemental Fig. 5, A and B) highlights MCF-7:5C cells’ proapoptotic UPR phase and programming to undergo apoptosis by 72 hours (Fig. 7E).

**Fig. 8.**
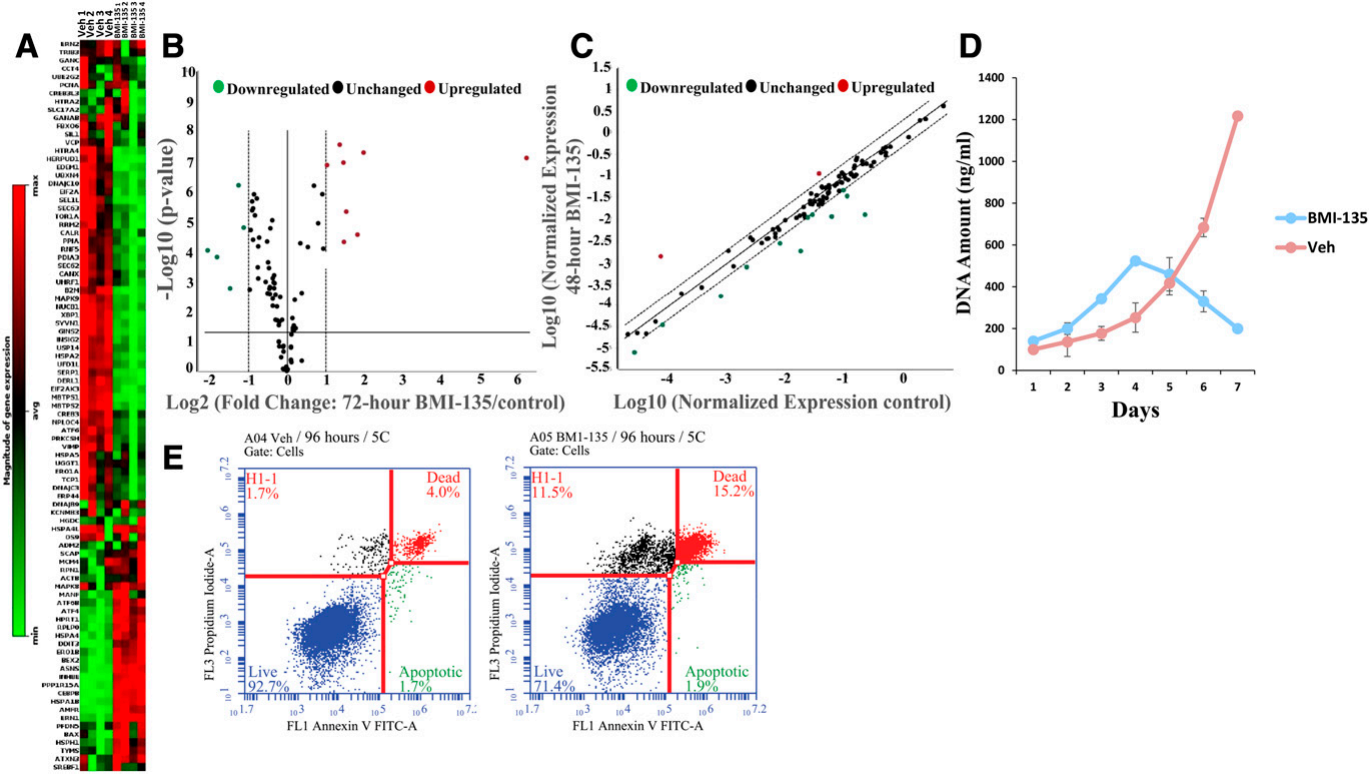
Human UPR RT^2^ PCR profiler PCR arrays, proliferation assays, and annexin V staining in MCF-7:5C cells with 48-hour, 72-hour, 96-hour, and 7-day BMI-135 treatments. (A) A heat map of 72-hour BMI-135 treatment, providing a visualization of the fold changes in expression between the select groups (from left to right; vehicle and BMI-135, respectively) for every gene in the array in the context of the array layout. (B) A volcano plot of 72-hour BMI-135 treatment. (C) A scatter plot of 48-hour BMI-135 treatment comparing the normalized expression of every gene on the array between the two select groups by plotting them against one another to quickly visualize large gene-expression changes. The central line indicates unchanged gene expression. The dotted lines indicate the selected fold-regulation threshold. Data points beyond the dotted lines in the upper left and lower right sections meet the selected fold-regulation threshold. (D) Effects of BMI-135 alone after 7 days of treatment. (E) Flow cytometry of 96-hour BMI-135 treatment. (B and C) Green represents downregulated, black unchanged, and red upregulated. Data are mean ± S.D. from three independent experiments performed in triplicate analyzed by one-way ANOVA. Veh, vehicle.

Cell viability and proliferation assays showed a decline in MCF-7:5C cell DNA amount with BMI-135 treatment by 96 hours (Fig. 8D). Furthermore, flow cytometry showed apoptosis by 96 hours (annexin staining 17.1% with BMI-135 vs. vehicle control 5.7%) (Fig. 8E). The time point of 72 hours was chosen to investigate the proapoptotic UPR gene regulation with BMI-135 treatment in MCF-7:5C cells, which preceded apoptosis by 96 hours. Another time point of 48 hours was chosen to compare and contrast the UPR gene regulation with that by 72 hours and show how this regulation is dynamic and culminates over time.

After 48-hour treatment with 1 μM BMI-135, the ERAD genes EDEM1 (*P* < 0.001), HTRA4 (*P* < 0.001), SYVN1 (*P* < 0.001), and HERPUD1 (*P* < 0.001) were downregulated (Fig. 8C; Supplemental Fig. 5C). The lipid metabolism genes MBTPS1 (*P* < 0.001) and SERP1 (*P* < 0.001) were downregulated (Fig. 8C; Supplemental Fig. 5C). By contrast, the genes CEBPB (*P* < 0.001) and INHBE (*P* < 0.001) were upregulated (Fig. 8C; Supplemental Fig. 5C). Interestingly, there was a 9.46-fold (*P* < 0.05) downregulation of EIF2AK3 (PERK) (Supplemental Fig. 5C), which might play a role in MCF-7:5C cells’ delayed course of apoptosis with BMI-135 treatment compared with E_2_ and E_4_. After a 72-hour treatment with 1 μM BMI-135, there was an intensified (or terminal) UPR gene regulation compared with 48 hours, with an upregulation of CEBPB (*P* < 0.001), INHBE (*P* < 0.001), PPP1R15A (GADD34, *P* < 0.001), DDIT3 (CHOP, *P* < 0.001), and ERN1 (IRE1*α*, *P* < 0.001). This is coupled with a downregulation of the ERAD genes, HTRA4 (*P* < 0.001), SEL1L (*P* < 0.01), and HERPUD1 (*P* < 0.001); the chaperone gene HSPA2 (*P* < 0.001); and the lipid metabolism gene MBTPS1 (*P* < 0.001) (Fig. 8B; Supplemental Fig. 5D).

The heat map of MCF-7:5C cells with BMI-135 treatment at 72 hours (Fig. 8A) displays a general UPR gene downregulation (situated on the right side of the heat map) compared with vehicle control (situated on the left). This general downregulation by 72 hours (Fig. 8B; Supplemental Fig. 5D) highlights MCF-7:5C cells’ trajectory to undergo apoptosis by 96 hours (Fig. 8E).

Cell viability and proliferation assays showed a decline in MCF-7:5C cell DNA amount with BPTPE treatment by day 8 (Supplemental Fig. 4D). Furthermore, flow cytometry showed apoptosis by day 8 (annexin staining 31.5% with BPTPE vs. vehicle control 9.4%) (Supplemental Fig. 4E). The time point of day 7 was chosen to investigate the proapoptotic UPR gene regulation, which precedes apoptosis by day 8. Another time point of day 3 was chosen to compare and contrast the UPR gene regulation with that of day 7 and show how this regulation is dynamic and culminates over time.

After a 3-day treatment with 1 μM BPTPE, there was a relatively minor UPR gene activation compared with the one seen by day 7 (Supplemental Figs. 4, B and C and 5, E and F). Interestingly, there was a 2.15-fold (*P* < 0.001) downregulation of EIF2AK3 with 3-day BPTPE treatment (Supplemental Fig. 5E), which might play a role in MCF-7:5C cells’ delayed course of apoptosis with BPTPE treatment compared with E_2_ and E_4_. This is also observed with BMI-135’s early treatment time point (Supplemental Fig. 5C). After a 7-day treatment with BPTPE, there was a downregulation of the ERAD gene HERPUD1 (*P* < 0.001), the lipid metabolism genes INSIG2 (*P* < 0.001) and MBTPS1 (*P* < 0.001), and the chaperone genes HSPA2 (*P* < 0.001) and DNAJB9 (*P* < 0.001) (Supplemental Figs. 4B and 5F).

The heat map of MCF-7:5C cells with BPTPE treatment at day 7 (Supplemental Fig. 4A) displays a general UPR gene downregulation (situated on the left side of the heat map) compared with vehicle control (situated on the right). This general downregulation by day 7 (Supplemental Figs. 4B and 5F) highlights MCF-7:5C cells’ programming to undergo apoptosis by day 8 (Supplemental Fig. 4E).

**Fig. 9.**
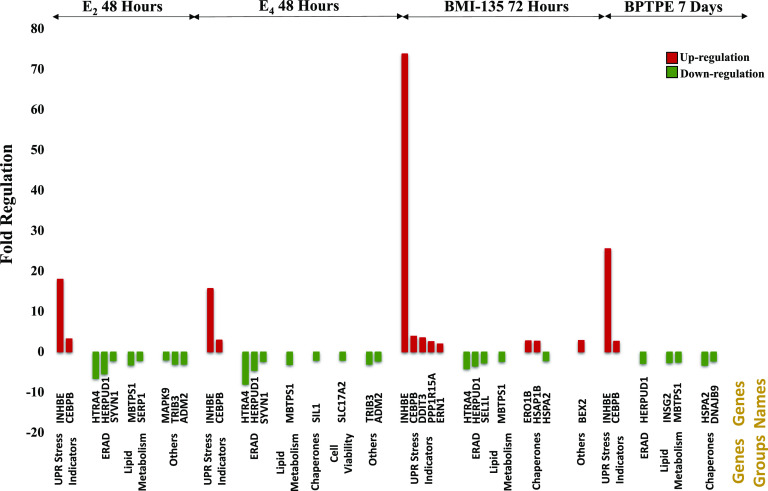
A schematic representation of the statistically significant UPR genes and their gene groupings with test compounds. The *y*-axis displays fold regulation, and the *x*-axis states the UPR genes and their groupings, demonstrating a signature proapoptotic UPR regulation at different time points with test compounds. Green represents downregulation and red upregulation. The ERAD proteins decrease cellular stress by degrading severely misfolded or unfolded proteins, and chaperones do so by folding the misfolded or unfolded proteins that could be rescued (Hetz, 2012). Lipid metabolism-related proteins play a critical role in lipid metabolism and homeostasis to combat cellular stress (Hetz and Saxena, 2017). The downregulation of these UPR gene groups (*P* < 0.05) as well as the upregulation of UPR stress indicators (INHBE and CEBPB) (*P* < 0.05) form a UPR phase whose regulation is characterized as terminal/proapoptotic (Maly and Papa, 2014; Grootjans et al., 2016).

The statistically significant regulated UPR genes with test compounds are stated and grouped at select time points (Fig. 9) to show the similar terminal UPR regulation preceding apoptosis.

#### E_4_ and BMI-135 Induce ThT Fluorescence as a Marker of UPR.

ThT has been successfully used for the detection and quantification of EnR stress and the UPR in living cells (Beriault and Werstuck, 2013) given that it directly interacts with the accumulated misfolded protein amyloid during the UPR (Beriault and Werstuck, 2013).

The “blue” Hoechst 33342 dye was used for counterstaining as a live cell nuclear dye (channel A), the “green” ThT dye was used as a UPR-indicative dye (channel B), and a colocalization of ThT and Hoechst 33342 dyes is shown (channel C). 17*β*-Estradiol and E_4_ were shown to induce ThT fluorescence by 48 hours, like the induction seen with positive control thapsigargin, and compared with vehicle control (Supplemental Fig. 6B). After 48-hour treatment, E_4_ had the highest ThT relative intensity/cell of 1.244892, and this was followed by thapsigargin of 0.875072; E_2_ of 0.741126; and BMI-135 of 0.497225, compared with vehicle control of 0.27594 (Table 2A).

**Fig. 10.**
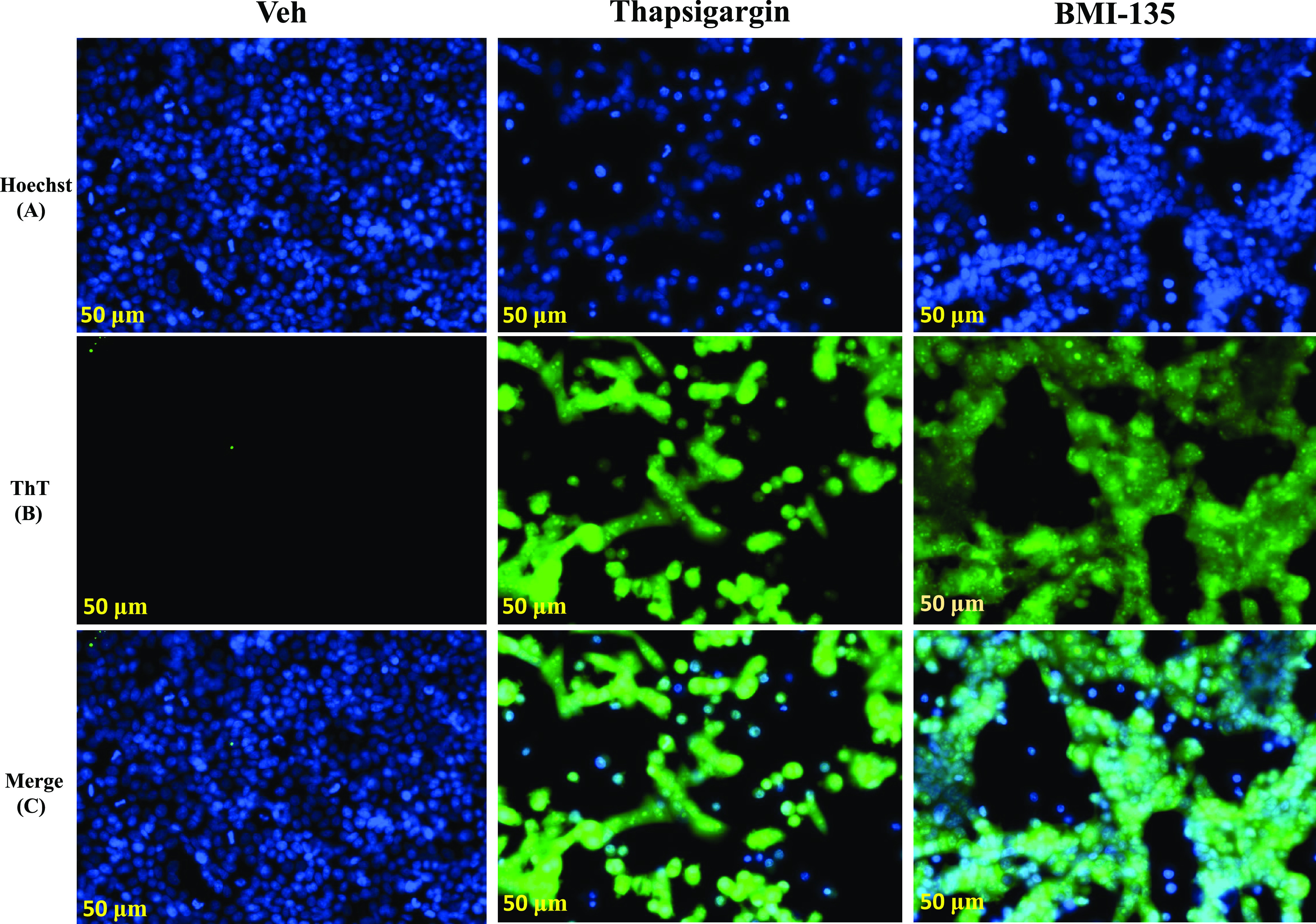
Detection of UPR in live MCF-7:5C cells using ThT fluorescent dye after 72-hour treatments as measured by the ZEISS Celldiscoverer 7 microscope. (A) Hoechst 33342 dye single panel (blue). (B) ThT dye single panel (green). (C) A merge or colocalization ThT + Hoechst 33342 dyes panel (blue and green). Treatments included 0.1% DMSO (vehicle control), 1 μM thapsigargin (positive control; promoting EnR stress by disrupting EnR Ca^2 +^ homeostasis), and 1 μM BMI-135. Scale bar, 50 μM. ThT relative intensity/cell, per treatment, is the mean of three biologic repeats with S.D. (Table 2B). Veh, vehicle.

BMI-135 induced a stronger delayed ThT fluorescence by 72 hours (Fig. 10B; Table 2B) compared with that seen by 48 hours (Supplemental Fig. 6B; Table 2A). The relative intensity/cell with 48-hour BMI-135 treatment was 0.497225 compared with vehicle control 0.27594 (Table 2A). However, the relative intensity/cell with 72-hour BMI-135 treatment was 4.878173 compared with vehicle control of 0.29573 (Table 2B). The relative intensity/cell over time is represented in Table 2.

#### E_4_ and BMI-135 Induce Apoptosis in Multiple Endocrine-Resistant and Estrogen-Independent BC Models.

Flow cytometry was used to determine whether the type of stress-induced cell death in MCF-7:5C, MCF-7:2A, and MCF-7:RAL cells was apoptosis when treated with 1 μM E_4_ and 1 μM BMI-135.

In MCF-7:5C, 1 μM E_4_ induced apoptosis (annexin staining 12.6% vs. vehicle control 4.5%) similar to the time course of 1 nM E_2_ (annexin staining 14.8% vs. vehicle control 4.5%) (Fig. 7E), which was by 72 hours. However, MCF-7:5C’s apoptosis with BMI-135 treatment (annexin staining 17.1% vs. vehicle control 5.7%) was delayed by 96 hours (Fig. 8E representing 96 hours; Supplemental Fig. 8D representing 72 hours). The antagonist 4OHT (as a negative control) and its pairing with E_2_, E_4_, and BMI-135 did not induce apoptosis by 72 or 96 hours, as predicted (unpublished data).

In MCF-7:2A, E_4_ induced apoptosis (annexin staining 6.7% vs. vehicle control 0.8%) similar to the time course of E_2_ (annexin staining 8% vs. vehicle control 0.8%) (Supplemental Fig. 8A), which was by day 9. However, MCF-7:2A’s apoptosis with BMI-135 treatment (annexin staining 7.3% vs. vehicle control 2.2%) was delayed by day 13 (Supplemental Fig. 8B representing day 13; Supplemental Fig. 8C representing day 9). The antagonist 4OHT (as a negative control) and its pairing with E_2_, E_4_, and BMI-135 did not induce apoptosis by day 9 or 13, as predicted (unpublished data).

In MCF-7:RAL, E_4_ induced apoptosis (annexin staining 7.6% vs. vehicle control 5.3%) similar to the time course of E_2_ (annexin staining 9% vs. control 5.3%) (Supplemental Fig. 9A), which was by day 14. However, MCF-7:RAL’s apoptosis with BMI-135 (annexin staining 8% vs. control 0.8%) was delayed until day 17 (Supplemental Fig. 9B representing day 17; Supplemental Fig. 9C representing day 14). The antagonists 4OHT and raloxifene and their pairing with E_2_, E_4_, and BMI-135 did not induce apoptosis by day 14 or 17, as predicted (Supplemental Fig. 9A). Interestingly, treatment of MCF-7:RAL cells with ICI for 3 weeks caused a decline in cell DNA amount (*P* < 0.05) (Supplemental Fig. 2C); however, this was not due to apoptosis (Supplemental Fig. 9D). Such observed effect of ICI in MCF-7:RAL could be attributed to growth inhibition by preventing cell replication.

#### Inhibition of PERK Pathway Blocks Apoptosis in MCF-7:5C with E_4_ and BMI-135 Treatments.

Blocking the UPR transducer PERK with 10 μM GSK G797800 in combination with 1 nM E_2_ and in combination with 1 μM E_4_ by 72 hours inhibited apoptosis (annexin staining 7.8% and 7.9%, respectively, vs. vehicle control 7%) (Supplemental Fig. 7A) compared with E_2_- and E_4_-alone treatments that trigger apoptosis (Fig. 7E) and compared with the negative control GSK G797800–alone treatment that does not trigger apoptosis (annexin staining 5.7% vs. vehicle control 7%) (Supplemental Fig. 7A).

**Fig. 11.**
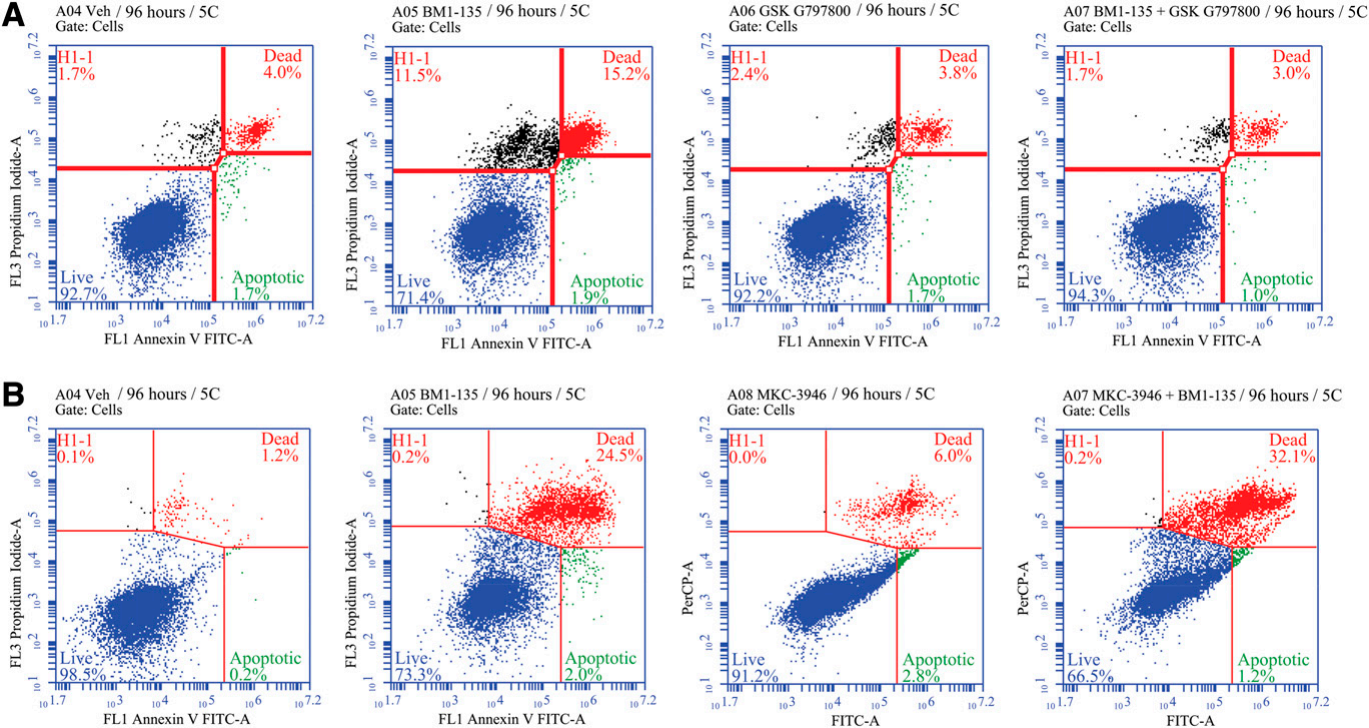
Flow cytometry in MCF-7:5C cells with BMI-135 plus a PERK inhibitor or an IRE1*α* inhibitor after 96-hour treatments. (A) MCF-7:5C cells were treated with 0.1% DMSO (vehicle control), 1 μM BMI-135, 10 μM GSK G797800, and 1 μM BMI-135 + 10 μM GSK G797800 then stained with annexin V–FITC and propidium iodide and analyzed by flow cytometry. Viable cells (left lower quadrant) are annexin V–FITC− and PI−; early apoptotic cells (right lower quadrant) are annexin V–FITC+ and PI−; dead cells (left upper quadrant) are PI+, and late apoptotic cells (right upper quadrant) are annexin V–FITC+ and PI+. An increased, late apoptotic effect is observed in the right upper quadrant. (B) MCF-7:5C cells were treated with 0.1% DMSO (vehicle control), 1 μM BMI-135, 20 μM MKC-3946, and 1 μM BMI-135 + 20 μM MKC-3946. Data are mean ± S.D. from three independent experiments analyzed by one-way ANOVA. (A and B).

Blocking PERK with 10 μM GSK G797800 in combination with 1 μM BMI-135 by 96 hours inhibited apoptosis (annexin staining 4% vs. vehicle control 5.7%) (Fig. 11A) compared with BMI-135–alone treatment that triggers apoptosis (Fig. 11A) and compared with GSK G797800–alone treatment (annexin staining 5.5% vs. control 5.7%) (Fig. 11A).

#### Inhibition of IRE1*α*:XBP1s Pathway Enhances Apoptosis in MCF-7:5C with E_4_ and BMI-135 Treatments.

The compound MKC-3946 inhibits IRE1*α* by inhibiting basal XBP1 splicing. Blocking the UPR transducer IRE1*α* with 20 μM MKC-3946 in combination with 1 μM E_4_ by 72 hours induces more apoptosis (annexin staining 34.1% vs. control 1.4%) (Supplemental Fig. 7B) compared with E_4_-alone treatment that triggers apoptosis (annexin staining 18.6% vs. control 1.4%) (Supplemental Fig. 7B) and compared with MKC-3946–alone treatment that triggers apoptosis (annexin staining 8.8% vs. control 1.4%) (Supplemental Fig. 7B).

Blocking IRE1*α* with 20 μM MKC-3946 in combination with 1 μM BMI-135 by 96 hours induces more apoptosis (annexin staining 33.3% vs. control 1.4%) (Fig. 11B) compared with BMI-135–alone treatment (annexin staining 26.5% vs. control 1.4%) (Fig. 11B) and compared with MKC-3946–alone treatment (annexin staining 8.8% vs. control 1.4%) (Fig. 11B).

## Discussion

**Fig. 12.**
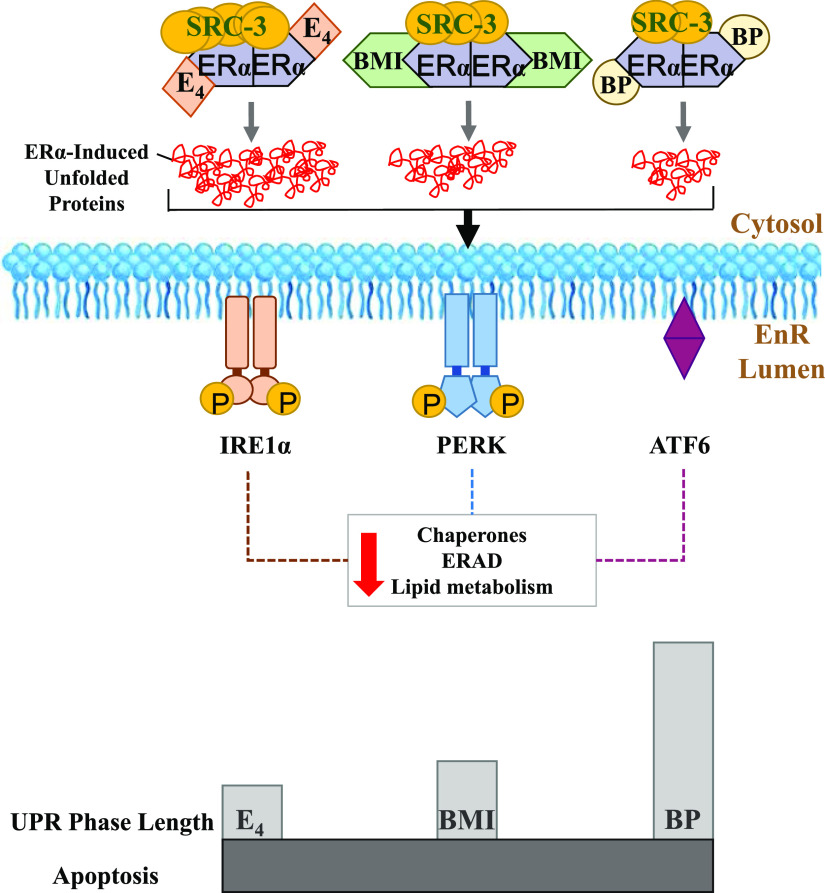
Schematic representation of the study’s concluded antitumor molecular mechanisms of E_4_, BMI-135, and BPTPE in LTED endocrine-resistant BC MCF-7:5C. Estetrol:ER*α* complex recruits the most coactivator SRC-3 and thus induces the most accumulation of unfolded proteins (highest threshold of stress), followed by BMI-135:ER*α* (BMI-135 is referred to as BMI in the illustration) and BPTPE:ER*α* (BPTPE is referred to as BP in the illustration). This differential ligand:ER*α*:coactivator-induced endoplasmic reticulum stress activates the transducers of the UPR, with a downregulation of chaperons, ERAD, and lipid metabolism genes and proteins (*P* < 0.05), which are considered prosurvival mechanisms. This downregulation state constitutes the proapoptotic UPR phase, which is reached quickly with E_4_, followed by BMI-135 and BPTPE, and eventually induces apoptosis.

Estetrol is a naturally occurring fetal estrogen, which is associated with a low risk of drug-drug interactions (CYP450 family) and a neutral impact on risk markers of venous thromboembolism (Singer et al., 2014; Coelingh Bennink et al., 2017; Verhoeven et al., 2018). BMI-135 is a member of a new class of estrogen mimics, which did not cause significant uterine proliferation (Molloy et al., 2014; Xiong et al., 2016). Estetrol and the ShERPA TTC-352 are currently being evaluated in endocrine-resistant MBC clinical trials (O’Regan et al., 2019; Schmidt et al., 2020). Our study, in a wide range of endocrine-resistant and estrogen-independent BC cell models as well as an endometrial cancer cell model, shows E_4_ and BMI-135 to be less potent full estrogen agonists (Figs. 2–5 and 6, B and F) with the induction of terminal UPR and apoptosis as their antitumor mechanism of action (Figs. 7–12; Supplemental Figs. 5, B and D and 6–9). Although BMI-135 exhibits a slightly delayed UPR-and-apoptosis biology compared with E_2_ and E_4_ (Figs. 7–11; Supplemental Figs. 6–9), it is still distinct from the much delayed UPR-and-apoptosis biology of the benchmark partial agonist BPTPE (Supplemental Fig. 4).

The application of long-term adjuvant endocrine therapy (Jordan et al., 1979) to treat ER-positive BC is invaluable for patient care. As a result, women’s lives are extended or saved (Early Breast Cancer Trialists’ Collaborative Group, 1998; Goss et al., 2003, 2005). Nonetheless, recurrence of endocrine-resistant stage IV BC is common (Pisani et al., 2002), hence the discovery of new therapeutic options remains a clinical priority.

Cell models (Pink et al., 1995; Liu et al., 2003; Lewis et al., 2005b; Ariazi et al., 2011; Fan et al., 2014) and athymic mice models (Gottardis and Jordan, 1988; Gottardis et al., 1989a,b; Yao et al., 2000) deciphered the evolution of acquired TAM resistance over years to eventually give rise to a vulnerability in BC: E_2_-induced apoptosis (Jordan, 2008; Jordan, 2015). Although estrogen is approved to treat BC, there is a reluctance to use estradiol as a salvage therapy in stage IV BC because of AEs. As a result, safer estrogenic alternatives are being considered.

Our goal was to compare and contrast the actions of E_4_ and BMI-135 with the well characterized partial agonist BPTPE. Our earlier pharmacological studies classified ER-binding ligands into agonists, partial agonists, and antagonists (Jordan, 1984; Jordan et al., 1984, 1986; Murphy et al., 1990a) and are essential to decipher the current molecular mechanisms of E_2_-induced apoptosis through the ER signal transduction pathway. These functional cell-based assays (Lieberman et al., 1983a,b; Jordan and Lieberman, 1984; Jordan et al., 1986) dovetailed with the subsequent X-ray crystallography studies of the agonist and antagonist ER complexes of the LBD (Brzozowski et al., 1997; Shiau et al., 1998). Our earlier biologic studies described E_2_-induced apoptosis (Jordan, 2015). Our current study shows that E_4_ and BMI-135:ER*α* complexes initiate and modulate the UPR (Figs. 7–12; Supplemental Figs. 5–7). This is an ER*α*-mediated (Supplemental Fig. 2) activation of the unfolded proteins’ synthesis and thus of cellular stress.

The intrinsic activity of the ER complex was evaluated by comparing and contrasting TFF1 and GREB1 estrogen-regulated gene activation with E_2_, BMI-135, BPTPE, and endoxifen treatments in WT MCF-7 and LTED MCF-7:5C cells (Fig. 3). The pharmacology of each ligand as a full agonist (E_2_, E_4_, and BMI-135) or a partial agonist (BPTPE) or an antagonist with no intrinsic activity (endoxifen) mirrored the pharmacology in cells (Fig. 2).

Molecular modeling studies demonstrated that E_4_, BMI-135, and BPTPE bind to the classic agonist conformation of ER*α*, similar to E_2_ (Fig. 6, A, C, and E). The flexible docking and MD simulations performed for BMI-135:ER*α* complex show the dynamic profile of the system to be similar to E_2_ (Supplemental Fig. 3A) with the ligand firmly bound to the active site (Supplemental Fig. 3B). Although BMI-135 is larger than E_2_, the same contacts have been observed, with the notable exception of the H-bond to His524 (Fig. 6B). These H-bonds and hydrophobic contacts are stable for both ligands, with slightly larger frequencies of occurrence with E_2_ (Supplemental Fig. 11, A and B), which indicates a stronger binding mode of E_2_. BPTPE exhibits equivalent binding contacts to E_2_ (Fig. 6, C and D) but forms a distinctive robust H-bond with Thr347 (Supplemental Fig. 11C), which induces the stability of the ligand binding but increases the mobility of H12 and the loop connecting H11 and H12, which affects the overall stability of the system. This is most likely responsible for the partial agonist profile of BPTPE. These data support the molecular classification of E_4_ and BMI-135 as full agonists and further explain their observed biologic behavior.

A comparison of E_4_, BMI-135, and BPTPE in multiple WT and LTED BC cell lines (Fig. 2; Supplemental Fig. 1) demonstrates the partial agonist actions of BPTPE on both growth (Figs. 2, A–E and 3, A and B; Supplemental Fig. 1, A–C and F) and E_2_-induced apoptosis (Figs. 2, F–H and 3, C and D; Supplemental Fig. 4, D and E). All experiments used BPTPE as a well characterized partial agonist (Jordan and Lieberman, 1984), which triggers delayed E_2_-induced apoptosis in LTED BC cells compared with E_2_ (Obiorah et al., 2014; Obiorah and Jordan, 2014) (Supplemental Fig. 4E). The mechanism is shown here to be through a delay in the induction of the proapoptotic UPR signaling (Supplemental Figs. 4, B and C and 5, E and F).

Delayed apoptosis with BPTPE (which contains a free para-hydroxyl on the phenyl ring) mirrors the delayed apoptosis with the synthesized angular triphenylethylene (TPE) derivative 3OH**TPE** (which contains the free para-hydroxyl) (Maximov et al., 2020). The other synthesized TPE derivative Z2OH**TPE** does not contain the free para-hydroxyl and causes early apoptosis, similar to E_2_ (Maximov et al., 2020). This free para-hydroxyl in BPTPE and 3OHTPE is part of the antiestrogenic side chain of endoxifen, which prevents the complete closure of ER*α*’s H12 over the ligand:LBD (Supplemental Fig. 11C). This delays the coactivators’ recruitment to the ER to form a transcriptionally active complex (Fig. 5B), which delays the ligand:ER*α*–induced transcription and translation of the unfolded proteins, resulting in delayed apoptosis (Supplemental Fig. 4).

Although BMI-135 does not exhibit the pharmacology of BPTPE (Figs. 2–5 and 6, A and B; Table 1), there is still a slight delay in the induction of the terminal UPR signaling and apoptosis, which is mediated by the BMI-135:ER*α* complex (Figs. 8, B and E and 10B; Supplemental Fig. 5D; Table 2B for the 72-hour time point vs. Fig. 8C; Supplemental Figs. 5C, 6B, and 8D; Table 2A for the 48-hour time point).

The ChIP assay (Fig. 5) is valuable in understanding the delayed apoptotic biology with BMI-135 and BPTPE. Earlier studies (Sengupta et al., 2013; Obiorah et al., 2014) demonstrated a reduction in the binding of the BPTPE:ER*α*:SRC-3 complex using the ChIP assay in MCF-7 cells, which is reproduced here (Fig. 5, A and B). A reduced DNA binding of the partial agonist complex occurs, which correlates with a reduction in the efficacy of the complex to synthesize misfolded or unfolded proteins, hence with a delay in the induction of the terminal UPR and apoptosis compared with E_2_ (Supplemental Fig. 4). Although BMI-135 recruits equivalent quantities of ER*α* (Fig. 5A), there is a reduced recruitment of the coactivator SRC-3 compared with E_2_ and E_4_. Nonetheless, BMI-135:ER*α*’s recruitment of SRC-3 is higher than that with BPTPE (*P* < 0.05) (Fig. 5B). This correlates with BMI-135’s slightly delayed induction of the terminal UPR and apoptosis (Fig. 8).

The downregulation of the prosurvival mechanisms, chaperones, ERAD, and lipid metabolism genes (*P* < 0.05), alongside the upregulation of marker UPR stress proteins (INHBE and CEBPB) (*P* < 0.05) constitute the terminal/proapoptotic UPR phase and underscore the antitumor mechanism of E_2_, E_4_, BMI-135, and BPTPE (Figs. 7–9 and 12; Supplemental Figs. 4 and 5).

Apoptosis with E_4_ and BMI-135 treatments was prevented by blocking the PERK pathway (Fig. 11A; Supplemental Fig. 7A). By contrast, blocking the IRE1*α*:XBP1s pathway after E_4_ and BMI-135 treatments enhanced apoptosis (Fig. 11B; Supplemental Fig. 7B). These data demonstrate the modulation of apoptosis with E_4_ and BMI-135 through the modulation of UPR’s subcellular sensors.

The timing of UPR-indicative ThT fluorescence with E_4_ and BMI-135 is synchronic with that of their proapoptotic UPR gene regulation (*P* < 0.05). The ThT fluorescence and terminal UPR gene regulation were shown to be by 48 hours with E_2_ and E_4_ (before apoptosis by 72 hours), by 72 hours with BMI-135 (before apoptosis by 96 hours), and by day 7 with BPTPE (before apoptosis by day 8) (Figs. 7, 8, and 10B; Supplemental Figs. 4, 5, and 6B; Table 2).

Translational research (Gottardis et al., 1988) identified a potential link between TAM treatment and the occurrence of endometrial cancer in patients (Jordan and Assikis, 1995). Raloxifene does not have an increased risk of endometrial cancer in clinical trials (Cummings et al., 1999; Vogel et al., 2006). BMI-135 is a raloxifene derivative (Fig. 1) (Xiong et al., 2016) and was tested to determine whether the ShERPA BMI-135:ER:coregulators complex is an agonist in the human endometrial cancer cell line Ishikawa transfected with 5x-ERE (Fig. 4). BPTPE exhibited a partial agonist activity (Fig. 4A), but both E_4_ and BMI-135 exhibited a less potent full agonist activity compared with E_2_ (Fig. 4A). This effect is mediated via the Ishikawa ER*α* (Fig. 4B). Although BMI-135 was shown not to induce uterine growth in a mouse xenograft model (Xiong et al., 2016), based on this study’s observations, it would be wise to require an endometrial screening for patients with BC receiving E_4_ or BMI-135.

Raloxifene induces acquired resistance as evidenced by SERM-stimulated BC cell growth (Liu et al., 2003; Balaburski et al., 2010) (Fig. 2H; Supplemental Figs. 1F and 2C). Such laboratory data have clinical significance because a case report of an antiestrogen withdrawal effect with raloxifene was reported (Lemmo, 2016). Raloxifene-resistant BC-stimulated growth has not been widely reported during the decades of treatment in patients with osteoporosis. This is surprising, but perhaps clinicians have not been aware of this form of SERM resistance. Nevertheless, our findings here (Supplemental Fig. 9, A and B) suggest that E_4_ or an ShERPA could be deployed after raloxifene discontinuation to induce tumor regression through apoptosis in raloxifene-resistant BC. Furthermore, ICI could be deployed, as we have shown here that it has a growth inhibitory effect (Supplemental Figs. 1F, 2C, and 9D).

Estrogen receptor-positive BC constitutes more than 70% of all BCs (Clark et al., 1984). Rosenberg and coworkers (2015) projected BC cases in the United States to double by 2030 compared with cases in 2011. The majority will be ER-positive BC. The development of new agents to treat ER-positive endocrine-resistant MBC remains a priority. Overall, the results of our work support the continuation of future clinical trials with the new agents E_4_ and ShERPAs.

## Abbreviations

AE: adverse event
ATF: activating transcription factor
BC: breast cancer
BPTPE: triphenylethylene bisphenol
ChIP: chromatin immunoprecipitation
CT: cycle threshold
DBPS: Dulbecco's phosphate-buffered saline
E_1_: estrone
E_2_: 17*β*-estradiol
E_3_: estriol
E_4_: estetrol
EnR: endoplasmic reticulum
ER: estrogen receptor
ERAD: EnR-associated degradation
ERE: estrogen-responsive element
FITC: fluorescein isothiocyanate
GREB1: Growth Regulation by Estrogen in Breast Cancer 1
ICI: ICI 182,780 fulvestrant
IRE1*α*: inositol-requiring enzyme 1
LBD: ligand-binding domain
LTED: long-term estrogen deprivation
MBC: metastatic BC
MBTPS1: Membrane Bound Transcription Factor Peptidase, Site 1
MD: molecular dynamics
4OHT: 4-hydroxyTAM
PCR: polymerase chain reaction
PERK: protein kinase regulated by RNA-like EnR kinase
PI: propidium iodide
Ralox: raloxifene
RMSD: root-mean-square deviation
RMSF: root-mean-square fluctuation
RT-PCR: real-time PCR
SERM: Selective Estrogen Receptor Modulator
ShERPA: Selective Human Estrogen Receptor Partial Agonist
SRC-3: steroid receptor coactivator 3
TAM: tamoxifen
TFF1: trefoil factor 1
ThT: Thioflavin T
TPE: triphenylethylene
UPR: unfolded protein response
WT: wild type
